# Nutritional and Metabolic Trajectories up to 10 Years After Metabolic and Bariatric Surgery: A Tertiary-Centre Retrospective Longitudinal Cohort Study

**DOI:** 10.3390/nu18193265

**Published:** 2026-10-03

**Authors:** Sebastian Mitchell, Priyanka Bhandari, Margaret Houston, Titisari Wardani, Nur Balzureen Suhaimi, Treeve Willcock, Brian Joyce, Gillian Drummond, Beverly Wallace, Kellyanne Mair, Frieda Whelan, Danielle Clyde, Anne Ewing, Peter J. Lamb, Andrew G. Robertson

**Affiliations:** Southeast Scotland Metabolic Bariatric Service, Clinical Department of Surgery, University of Edinburgh, Royal Infirmary of Edinburgh, 51 Little France Crescent, Edinburgh EH16 4SA, UK; sebastian.mitchelll@doctors.org.uk (S.M.); priyanka.bhandari2@nhs.scot (P.B.); margaret.houston@nhs.scot (M.H.); titisari.wardani@nhs.scot (T.W.); work.balzureensuhaimi@gmail.com (N.B.S.); treeve.willcock@nhs.scot (T.W.); brian.joyce@nhs.scot (B.J.); gillian.drummond@nhs.scot (G.D.); beverley.wallace@nhs.scot (B.W.); kellyanne.mair@nhs.scot (K.M.); frieda.whelan@nhs.scot (F.W.); danielle.clyde@nhs.scot (D.C.); anne.ewing@nhs.scot (A.E.); peter.lamb@nhs.scot (P.J.L.)

**Keywords:** metabolic & bariatric surgery, micronutrient deficiency, sleeve gastrectomy, Roux-en-Y gastric bypass, secondary hyperparathyroidism, vitamin D, anaemia, trace elements, longitudinal follow-up

## Abstract

Background/Objectives: Metabolic and bariatric surgery (MBS) is the most effective durable treatment for severe obesity but predisposes to micronutrient deficiency. Long-term, broad-panel biochemical data are scarce. We examined nutritional and metabolic trajectories over up to 10 years and compared sleeve gastrectomy (SG) with Roux-en-Y gastric bypass (RYGB). Methods: Retrospective single-centre cohort undergoing MBS (NHS Lothian, 2011–2025). Twenty-five biochemical markers were captured at baseline, 6 months, 1, 5 and 10 years, and analysed using linear mixed-effects models adjusted for procedure, sex, age, baseline BMI, diabetes and deprivation, with Benjamini-Hochberg correction. Results: Of 458 patients, 426 were analysed (82.4% female; median age 49 years; median baseline BMI 44.2 kg/m^2^; SG 55.2%, RYGB 41.1%; median follow-up 7.0 years). Median total and excess weight loss were 21.6% and 49.2%. Pre-operative type 2 diabetes affected 99/422 (23.5%), resolving in 41 (41.4%). Adjusted calcium fell (*p* < 0.001) and parathyroid hormone (PTH) rose (+0.22 pmol/L/year [95% CI 0.11, 0.33], *p* < 0.001) despite vitamin D increasing (+1.20 nmol/L/year, *p* < 0.001); elevated PTH persisted in ~33%. Haemoglobin declined (−0.32 g/L/year, *p* = 0.026), below-range values rising from 26.3% (109/415) to 38.5% (84/218). Serum iron and transferrin saturation rose (*p* = 0.034; *p* = 0.002). B-vitamins increased (all *p* < 0.001) except thiamine. Selenium, red-cell selenium and zinc fell (all *p* < 0.05); albumin declined (*p* < 0.001); cholesterol was stable. Folate and vitamin E trajectories differed between SG and RYGB; other markers did not. The PTH rise was steeper with proton pump inhibitor use (*p* = 0.002) and baseline BMI ≥ 50 kg/m^2^ (*p* = 0.003). Conclusions: Within a structured service with high supplement compliance, frank deficiency was uncommon for most markers. Nonetheless, progressive biochemical changes emerged and persisted, most consistently falling calcium with rising PTH and declining haemoglobin. These findings support indefinite, systematic nutritional surveillance after MBS.

## 1. Introduction

Obesity is one of the major global health challenges of this century. In 2022, more than 890 million adults were living with obesity worldwide, with prevalence having more than doubled in women and nearly tripled in men since 1990 [1,2]. Metabolic and bariatric surgery (MBS) is the most effective durable treatment for severe obesity, producing substantial and sustained weight loss, improvement or remission of type 2 diabetes, and reductions in long-term mortality [3,4,5]. More than half a million procedures are performed internationally each year, with sleeve gastrectomy (SG) and Roux-en-Y gastric bypass (RYGB) accounting for the large majority [6]. These benefits are accompanied by important nutritional consequences. Reduced dietary intake, changes in gastric acid exposure and gastrointestinal transit, and, after RYGB, bypass of the proximal small bowel can impair nutrient intake and absorption [7,8]. Deficiencies of iron, vitamin B12, folate, vitamin D and calcium, together with secondary hyperparathyroidism and metabolic bone disease, are well recognised and form a central part of postoperative nutritional surveillance [9,10,11].

Despite the predominance of SG and RYGB, their long-term nutritional effects remain incompletely characterised. Most evidence is concentrated within the first few postoperative years, while studies reporting detailed biochemical outcomes beyond five years are considerably fewer [12,13]. Long-term deficiencies nevertheless remain clinically important. In one multicentre cohort, 80.7% of patients had at least one nutritional deficiency eight years after surgery despite reported adherence to supplementation [14]. Evidence is particularly limited for trace elements such as zinc, copper, selenium and manganese, which are measured and reported far less consistently than iron, vitamin B12 or vitamin D [15]. Long-term direct comparisons of SG and RYGB have also tended to examine selected nutritional outcomes rather than changes across a broad biochemical panel [12,16]. The extent to which nutritional abnormalities persist or emerge over longer follow-up, and whether their trajectories differ between procedures, therefore, remains uncertain.

We therefore aimed to characterise nutritional and metabolic changes after MBS in a single-centre retrospective longitudinal cohort with follow-up of up to 10 years. We examined biochemical trajectories and the prevalence of abnormal results over time across a broad nutritional panel and compared longitudinal patterns between SG and RYGB.

## 2. Materials and Methods

### 2.1. Study Design and Setting

This was a retrospective analysis of a prospectively maintained single-centre MBS database, including adults who underwent MBS at NHS Lothian and were followed in the local bariatric clinic between January 2011 and December 2025. NHS Lothian serves a resident population of approximately 800,000 across Edinburgh and the lothians [17]. Patients were followed as part of routine post-operative care.

### 2.2. Study Population

All adults (≥18 years) undergoing MBS at NHS Lothian during the study period were eligible, comprising SG, RYGB and adjustable gastric band (AGB); a small number of revisional procedures (e.g., band to bypass conversion) were included. Patients were excluded if no clinical record was available or if no biochemical results were recorded at any timepoint. All eligible patients contributed to the descriptive and pooled analyses. Baseline BMI reflected weight at the time of surgery. In a small number of patients this was below conventional surgical thresholds, either because of mandatory pre-operative weight loss (a minimum of 5% was required, so referral BMI was higher) or, in revisional cases, because the recorded weight followed earlier bariatric surgery.

### 2.3. Procedure Selection

From 2010, patients completed a 12-week intensive pre-operative information course, including consultations with dietitians, psychologists and bariatric specialist nurses. At least 5% weight loss was required before full surgical assessment. Patients were offered a choice of SG or RYGB. Patients with T2DM, severe reflux or a higher BMI were encouraged towards RYGB. Smokers, patients on complex medications and those with complex previous abdominal surgery were directed towards SG. Patients were counselled that SG would be performed if technical difficulties arose intra-operatively. AGB was performed in a small number of patients following the same assessment pathway.

### 2.4. Surgical Technique

All procedures were performed laparoscopically using a standardised unit technique, which remained unchanged over the study period. For SG, the stomach was mobilised from 5 cm proximal to the pylorus to the left crus, calibrated over a 34 Fr tube and divided with 60 mm Endo-GIA staplers (Medtronic, Minneapolis, MN, USA), with the lower staple line oversewn using 2/0 PDS. For RYGB, the gastric pouch was created below the second vessel using 45 mm and 60 mm Endo-GIA staplers. A 50 cm biliopancreatic limb and a 150 cm Roux limb were fashioned, with an antecolic, antegastric stapled gastrojejunostomy and a stapled jejunojejunostomy. Jejunojejunal and Petersen’s defects were closed. AGB was performed in a small number of patients using a standard laparoscopic technique.

### 2.5. Data Collection

Data were extracted from the local bariatric database and the TRAK electronic health record (NHS Lothian, Edinburgh, UK). Biochemical results were obtained from the TRAK laboratory system, all processed by Edinburgh and Lothian Laboratory Medicine (NHS Lothian). Recorded variables included demographic, operative, anthropometric and comorbidity data. This included age, sex, baseline body mass index (BMI), pre-operative type 2 diabetes mellitus (T2DM) and Scottish Index of Multiple Deprivation (SIMD) quintile; together with supplement prescription and patient-reported adherence. Supplements were prescribed according to a standard post-operative regimen based on BOMSS guidance, comprising a multivitamin-mineral preparation (Forceval, Wiltshire, UK), with calcium and vitamin D, vitamin B12, ferrous fumarate and vitamin K prescribed when indicated [10]. This approach was unchanged over the study period. Nutritional markers were assessed at pre-operative baseline and at 6 months, 1, 5 and 10 years post-operatively, in line with the routine follow-up schedule recommended by the British Obesity and Metabolic Surgery Society (BOMSS) [10]. For each timepoint, the biochemical result nearest to the scheduled date was used, within a ±1-month window at 6 months and 1 year, a ±3-month window at 5 and 10 years. As recruitment spanned January 2011 to December 2025, follow-up duration varied by operative date, and patients operated more recently had not yet reached the later timepoints. The number of patients contributing a result therefore varied by marker and declined with time; the denominator is reported for every estimate.

### 2.6. Biochemical Markers and Reference Ranges

Twenty-five nutritional and metabolic markers were analysed, grouped by physiological system, and classified against the Edinburgh and Lothian Laboratory Medicine reference ranges [18]. Bone and mineral markers comprised vitamin D (50–139 nmol/L; deficiency < 50), adjusted calcium (2.2–2.6 mmol/L), phosphate (0.8–1.4 mmol/L), magnesium (0.7–1.0 mmol/L), parathyroid hormone (PTH) (1.6–6.9 pmol/L) and alkaline phosphatase (40–125 U/L). Iron and anaemia markers comprised haemoglobin (130–180 g/L), ferritin (20–300 µg/L), iron (14–32 µmol/L), transferrin (2–4 g/L) and transferrin saturation (20–50%). B-vitamins and haematinics comprised vitamin B12 (180–2000 ng/L; deficiency < 180), folate (2.8–20 µg/L; deficiency < 2.8), vitamin B1 (thiamine) (275–675 nmol TDP/g Hb; deficiency < 275), vitamin B2 (1.0–3.4 nmol FAD/g Hb; deficiency < 1.0) and vitamin B6 (250–680 pmol/g Hb, with additional thresholds for over-supplementation >2000 and toxicity >4000). Fat-soluble vitamins comprised vitamin A (1.0–3.0 µmol/L; deficiency < 1.0, severe ≤ 0.7, toxicity > 4.5) and vitamin E (12–46 µmol/L). Trace elements comprised zinc (11–18 µmol/L, with frank deficiency defined as <6 µmol/L), copper (10–22 µmol/L), selenium (0.75–1.50 µmol/L), red-cell selenium (3.0–9.0 nmol/g Hb) and manganese (70–280 nmol/L). General and metabolic markers comprised albumin (36–47 g/L) and total cholesterol, the latter reported descriptively with no deficiency threshold. For each marker, the direction of abnormality (low, high, or both) was pre-specified; values were classified as outside the reference range accordingly, and where separate clinical deficiency thresholds are stated above, this defined deficiency.

### 2.7. Outcomes

This was a descriptive study of longitudinal nutritional and metabolic outcomes, without a single primary endpoint; all 25 markers were assessed as co-equal outcomes, with multiplicity addressed statistically. For each marker, three outcomes were evaluated: the trajectory of concentration over follow-up (annual rate of change), the proportion of patients outside the reference range at each timepoint, and any difference in trajectory between SG and RYGB.

### 2.8. Statistical Analysis

Continuous variables were summarised as median [interquartile range; IQR] and categorical variables as *n* (%), calculated on non-missing denominators. Between-procedure comparisons were restricted to SG and RYGB, as AGB and other procedures were too few to support reliable comparison; these patients were retained in all other analyses. Each marker was modelled using a linear mixed-effects model with a random intercept per patient to account for repeated measures; a random-intercept structure was used throughout. Fixed effects comprised time (years from surgery), an interaction between time and procedure (SG vs. RYGB), and adjustment for sex, age at surgery, baseline BMI, pre-operative T2DM and SIMD quintile. Time was modelled both as a continuous term, estimating a constant annual rate of change, and, in a companion model, as a categorical factor, estimating the change from baseline at each timepoint. Pooled estimates are presented because all models included a procedure × time interaction, which formally tested whether trajectories differed between SG and RYGB. Degrees of freedom used the Satterthwaite approximation. *p*-values were corrected for multiple comparisons using the Benjamini-Hochberg false discovery rate within each of the six marker families defined above. Four pre-specified subgroup and sensitivity analyses were performed across all markers to assess effect modification and robustness: by proton pump inhibitor (PPI) use, by baseline BMI (<50 vs. ≥50 kg/m^2^), restricted to patients’ adherent to all prescribed supplements, and excluding patients with coexisting gastrointestinal pathology. PPI use was selected because gastric acid suppression may impair calcium absorption, and PPI use has been associated with hyperparathyroidism [19]. PPI use was recorded as a binary patient-level variable (prescribed or not) and was not captured at individual timepoints. Analyses were performed in R version 4.5.2 (packages lme4, lmerTest and emmeans).

Models were fitted to all available observations without imputation and assumed data were missing at random conditional on the covariates, an approximately linear change over time, and normally distributed residuals; the companion categorical-time model was fitted to characterise non-linear trajectories. Adherence in the sensitivity analysis was defined at patient level as patient-reported adherence to all prescribed supplements.

### 2.9. Ethics and Governance

This work was undertaken as part of continuous clinical audit of routinely collected data and, under the UK Health Research Authority framework, did not meet the criteria for research; it therefore did not require Research Ethics Committee approval or individual patient consent. The study was conducted in accordance with the Declaration of Helsinki. All data were anonymised and held in a password-protected repository on NHS Lothian servers, in accordance with local information-governance policy.

## 3. Results

### 3.1. Cohort Characteristics

**Figure 1:**
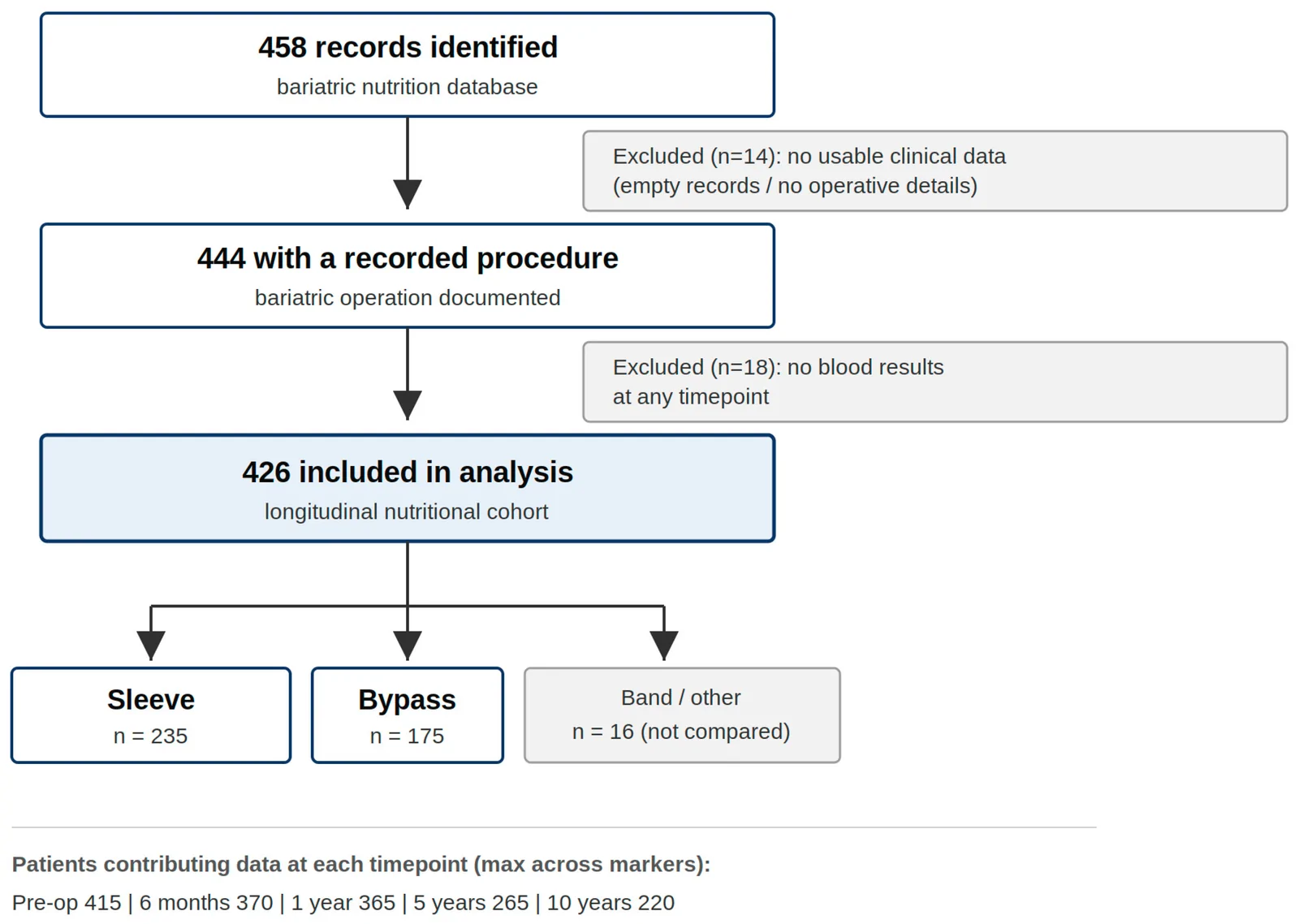
Flow of participants through the study, from records identified to patients included in the analysis cohort, with reasons for exclusion.

Of 458 records identified, 32 were excluded (14 for absent clinical data, 18 for no blood results at any timepoint), leaving 426 patients for analysis (Figure 1).

The cohort was 82.4% female (351/426), with median age at surgery 49 years [IQR 40–55] and median baseline BMI 44.2 kg/m^2^ [39.9–49.6]. Procedures were SG (235/426, 55.2%), RYGB (175/426, 41.1%), AGB (12/426, 2.8%) and other (4/426, 0.9%; three gastric band removals and one gastric bypass revision). Median %TWL was 21.6% [12.2–31.1] (*n* = 416) and median %EWL 49.2% [27.9–72.6] (*n* = 408), with 245/408 (60.0%) achieving >50% excess weight loss. Pre-operative T2DM was present in 99/422 (23.5%), of whom 41 (41.4%) resolved. In total, 218/406 (53.7%) fell in the two most deprived SIMD quintiles. Supplementation was near-universal (Forceval 386/424, 91.0%; PPI 277/424, 65.3%), and 55/426 (12.9%) had coexisting gastrointestinal pathology. Median follow-up was 7.0 years [2.2–11.0] (Table 1).

### 3.2. Bone and Mineral Metabolism

**Figure 2:**
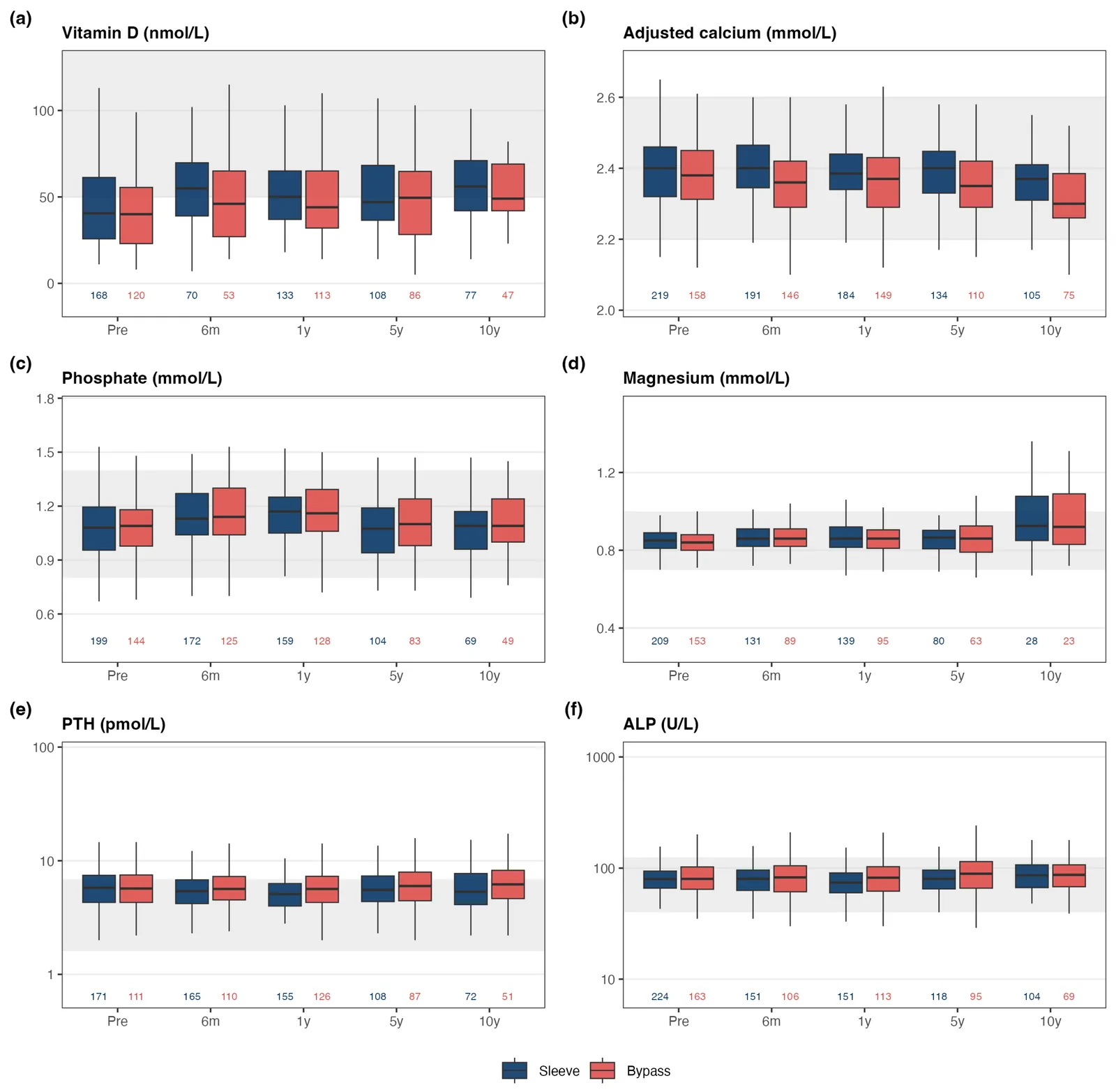
Bone and mineral markers by procedure across timepoints. (**a**) Vitamin D; (**b**) adjusted calcium; (**c**) phosphate; (**d**) magnesium; (**e**) parathyroid hormone; (**f**) alkaline phosphatase. Shaded bands indicate reference ranges. Numbers beneath each box indicate the number of patients measured at that timepoint.

Vitamin D deficiency affected 66.3% (197/297) pre-operatively and 40.8% (51/125) at 10 years, with concentrations rising over follow-up (+1.20 nmol/L/year [95% CI 0.53, 1.86], *p* < 0.001, *n* = 374). Adjusted calcium fell (−0.004 mmol/L/year [−0.006, −0.002], *p* < 0.001, *n* = 407), parathyroid hormone rose (+0.22 pmol/L/year [0.11, 0.33], *p* < 0.001, *n* = 392; elevated in 29.8% [87/292] pre-operatively and 32.8% [41/125] at 10 years), and alkaline phosphatase rose (+1.17 U/L/year [0.02, 2.32], *p* = 0.045, *n* = 402). Phosphate fell (−0.007 mmol/L/year [−0.012, −0.002], *p* = 0.009, *n* = 383) and magnesium rose (+0.008 mmol/L/year [0.003, 0.014], *p* = 0.004, *n* = 392), both within range in >93% of measurements. No bone marker differed significantly between procedures (Table 2 and Table 3, Figure 2, Supplementary Tables S1 and S2).

### 3.3. Iron Status and Anaemia

**Figure 3:**
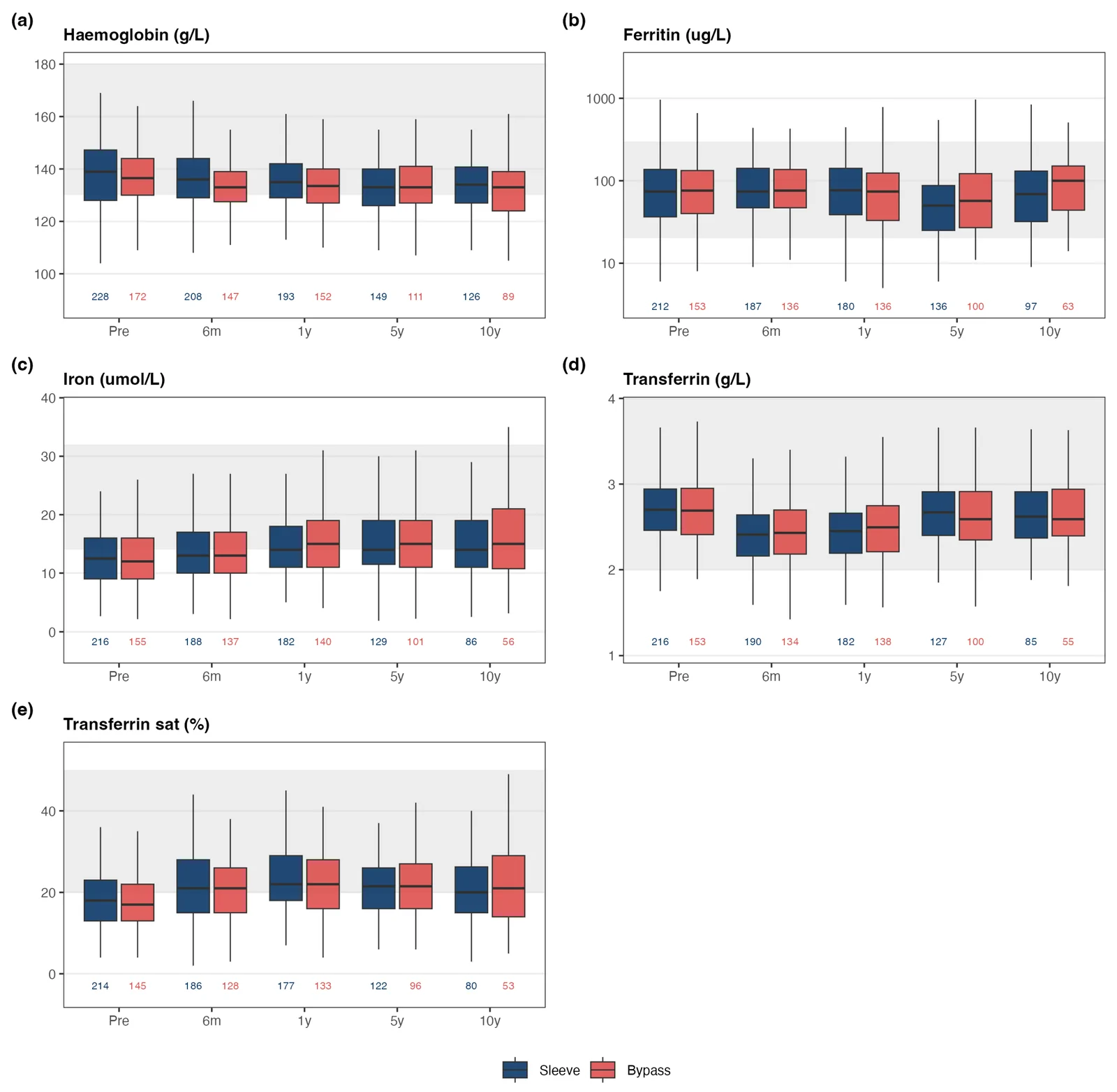
Iron status and anaemia markers by procedure across timepoints. (**a**) Haemoglobin; (**b**) ferritin; (**c**) iron; (**d**) transferrin; (**e**) transferrin saturation. Shaded bands indicate reference ranges. Numbers beneath each box indicate the number of patients measured at that timepoint.

Haemoglobin declined (−0.32 g/L/year [−0.57, −0.08], *p* = 0.026, *n* = 408), with the proportion below range rising from 26.3% (109/415) to 38.5% (84/218) at 10 years. Serum iron rose (+0.25 µmol/L/year [0.04, 0.46], *p* = 0.034, *n* = 402) and transferrin saturation rose (+0.46%/year [0.20, 0.72], *p* = 0.002, *n* = 390), the proportion with low saturation falling from 59.0% (217/368) to 45.9% (62/135). Ferritin increased on average (+4.17 µg/L/year [0.32, 8.01], *p* = 0.042, *n* = 404) though individual timepoint contrasts were non-significant; transferrin did not change (*p* = 0.113). No iron marker differed between procedures (Table 2 and Table 3, Figure 3, Supplementary Table S2).

### 3.4. B-Vitamins and Haematinics

**Figure 4:**
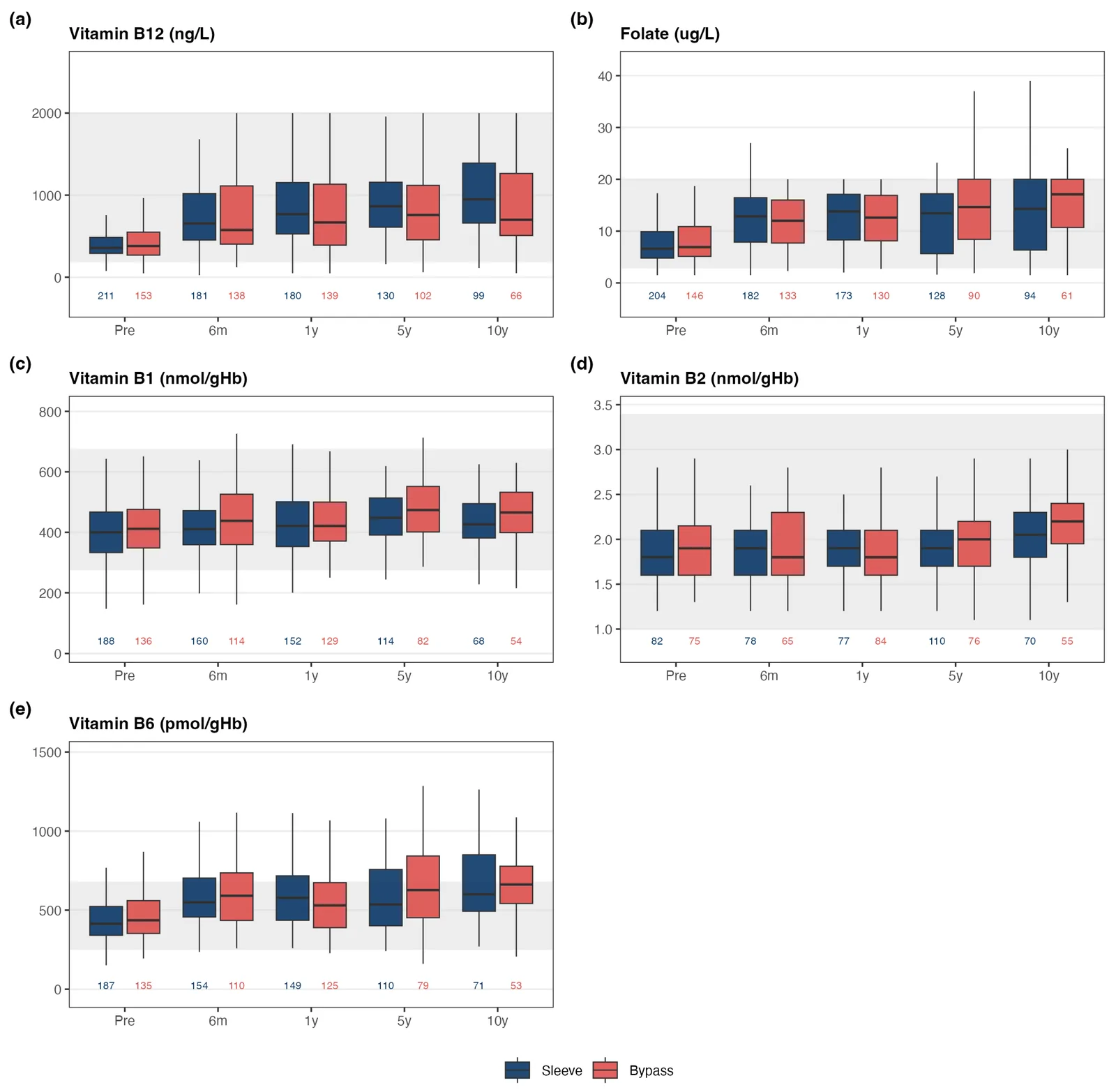
B-vitamin and haematinic markers by procedure across timepoints. (**a**) Vitamin B12; (**b**) folate; (**c**) thiamine (vitamin B1); (**d**) vitamin B2; (**e**) vitamin B6. Shaded bands indicate reference ranges. Numbers beneath each box indicate the number of patients measured at that timepoint.

Vitamin B12 (+29.1 ng/L/year [15.6, 42.6], *p* < 0.001, *n* = 402), folate (+0.61 µg/L/year [0.43, 0.78], *p* < 0.001, *n* = 398), vitamin B2 (*p* < 0.001, *n* = 359) and vitamin B6 (+13.7 pmol/gHb/year [6.4, 21.0], *p* < 0.001, *n* = 394) rose over follow-up; thiamine (B1) did not change significantly (+3.08 nmol/gHb/year [−0.46, 6.63], *p* = 0.088, *n* = 388). Values below range were uncommon throughout. Folate rose less after SG than after RYGB (−0.32 µg/L/year [−0.54, −0.10], *p* = 0.026) (Table 2 and Table 3, Figure 4, Supplementary Table S2).

### 3.5. Fat-Soluble Vitamins, Trace Elements and Cholesterol

Vitamin A rose marginally (+0.019 µmol/L/year [0.003, 0.035], *p* = 0.036, *n* = 365) and vitamin E rose more after SG than after RYGB (+0.81 µmol/L/year [0.39, 1.23], *p* < 0.001, *n* = 327). Zinc fell (−0.075 µmol/L/year [−0.129, −0.022], *p* = 0.015, *n* = 382) and was below the reference range in 45.1% (526/1167) of measurements, though frank deficiency (<6 µmol/L) occurred in 0.4%. Selenium (−0.010/year [−0.018, −0.002], *p* = 0.033, *n* = 343) and red-cell selenium (−0.097/year [−0.155, −0.039], *p* = 0.005, *n* = 341) fell, low selenium rising from 1.3% (2/153) to 14.0% (17/121). Copper and manganese did not change (*p* = 0.351; *p* = 0.280). Albumin declined (−0.226 g/L/year [−0.307, −0.146], *p* < 0.001, *n* = 387), the proportion below range rising from 13.4% (49/367) to 42.8% (68/159). Total cholesterol was stable (4.5 mmol/L [4.0–5.3] pre-operatively, 4.6 [3.8–5.4] at 10 years; *p* = 0.482, *n* = 375). (Supplementary Table S3) (Supplementary Figures S1–S3).

### 3.6. Subgroup and Sensitivity Analyses

Subgroup and sensitivity analyses were performed for all 25 markers across six strata. The principal between-stratum differences were in PTH: the increase was greater with a PPI (+0.31 pmol/L/year [0.13, 0.49], *p* = 0.002) than without (+0.13 [0.01, 0.25], *p* = 0.062), and greater with baseline BMI ≥ 50 kg/m^2^ (+0.60 [0.25, 0.95], *p* = 0.003) than <50 (+0.10 [0.00, 0.20], *p* = 0.070). In the compliant-only analysis, vitamin D and haemoglobin trajectories were unchanged while the PTH slope attenuated (+0.06 [−0.03, 0.16], *p* = 0.200); excluding patients with gastrointestinal pathology did not materially alter any estimate. For marker-by-marker analyses see Supplementary Table S4.

## 4. Discussion

Overall, supplement compliance was high, and frank deficiency was uncommon for most markers, but progressive changes in biomarker concentrations were evident over up to 10 years of follow-up. Followed for up to 10 years after MBS, the dominant finding was progressive disturbance of the bone-mineral axis adjusted calcium fell, PTH and alkaline phosphatase rose, and elevated PTH persisted in roughly a third of patients. This was despite a concurrent rise in serum vitamin D and near-universal supplementation. Haemoglobin declined gradually, several trace elements and albumin fell, B-vitamins rose, and cholesterol remained stable. Deficiencies therefore emerged and persisted even within a closely monitored, well-supplemented population.

The bone-mineral changes accord with longitudinal data showing PTH and secondary hyperparathyroidism (SHPT) rising after MBS, reaching around 38% prevalence at five years [20]. The distinguishing feature of our cohort is that PTH rose even as vitamin D increased. This dissociation suggests that the hyperparathyroid drive is not simply a consequence of low vitamin D but may also be sustained by calcium malabsorption from the bypassed or reconfigured proximal small bowel. Repleting serum vitamin D may therefore not by itself normalise parathyroid activity, consistent with reports that SHPT persists in a substantial minority even when vitamin D is adequate [20]. The clinical importance of this lies in bone: fracture risk after gastric bypass rises with time and is not attenuated by the degree of calcium and vitamin D supplementation, implying that biochemical correction alone may be an insufficient surrogate for skeletal protection [11]. Consistent with this, a long-term study with densitometry found that bone metabolism markers remained largely within reference ranges while bone mineral density fell and fracture risk rose across SG, RYGB and one-anastomosis gastric bypass (OAGB) [21]. Pooled data likewise show vitamin D deficiency increasing with follow-up, from 35% within one year to over 50% beyond five [22]. This underscores that the bone-mineral axis requires active, indefinite surveillance rather than one-off correction.

Haemoglobin fell and the proportion below range rose from a quarter to over a third, mirroring meta-analytic data in which post-bypass anaemia rises from 15% within one year to 35% beyond five [23]. A notable feature of our data is that haemoglobin declined while serum iron and transferrin saturation rose. This apparent paradox may reflect iron supplementation raising circulating indices, together with ferritin behaving as an acute-phase reactant, superimposed on a slow attritional anaemia that is likely multifactorial, reflecting chronic blood loss, incomplete iron repletion, and other haematinic deficiencies. These markers should therefore be interpreted together rather than in isolation [16,23]. Consistent with this, the SLEEVEPASS trial reported iron deficiency in 41% after RYGB versus 14% after SG at 10 years; although iron status is the domain in which procedure choice is most consistently reported to matter, we found no significant procedural difference, possibly reflecting our uniform supplementation protocol [12].

The remaining marker families moved largely as supplementation would predict. Vitamin B12, folate, vitamin B2 and vitamin B6 rose, while thiamine was unchanged, indicating that oral replacement of the commonly supplemented vitamins was broadly effective. Trace elements, by contrast, diverged: selenium, red-cell selenium and zinc declined, whereas copper and manganese held; frank zinc deficiency was rare, most low values sitting just below the reference range. Albumin declined markedly. This dissociation, supplemented vitamins maintained, but trace elements and albumin drifting downward, reflects reports that trace elements are both under-monitored and prone to late decline, and that deficiencies persist despite supplement use [14,15]. It suggests that standard multivitamin-mineral regimens may not fully cover the trace-element requirement over the long-term.

A central feature of our cohort is that these changes occurred within a service providing regular, extended nutritional follow-up and near-universal supplementation. That deficiencies nonetheless emerged reinforces, rather than undermines, the case for lifelong monitoring: even close supervision does not eliminate risk, and abnormalities persisted among adherent patients. In the compliant-only analysis, vitamin D and haemoglobin trajectories were unchanged, whereas the rise in PTH was attenuated and no longer significant. This suggests that adherence may modify the bone-mineral response, although the analysis was not designed to test this directly [9,10]. This echoes a multicentre cohort in which 80.7% of patients had at least one deficiency at eight years despite good adherence [14]. Two subgroups were at particular risk of bone-mineral disturbance: the PTH rise was steeper in patients taking PPIs and in those with a higher baseline BMI. The former is biologically plausible, as acid suppression impairs the solubilisation and intestinal absorption of calcium and may thereby amplify the hyperparathyroid response; an effect reported even among PPI users taking calcium supplements [19]. The latter may reflect the lower circulating vitamin D characteristic of greater adiposity, attributed to volumetric dilution and sequestration within a larger fat mass, together with a greater malabsorptive burden [24]. Both identify patients in whom closer biochemical surveillance, and review of PPI use, may be warranted.

To our knowledge, this is among the few studies to report outcomes across a 25-marker panel. This includes trace elements rarely captured elsewhere, over up to 10 years of follow-up after MBS, analysed with mixed-effects models that use all available longitudinal measurements. Most existing evidence is short-term, anaemia-focused, or confined to the commonly measured vitamins [16]. By describing the full biochemical trajectory within a single, protocol-consistent service, our data complement randomised evidence such as SLEEVEPASS, whose controlled design maximises internal validity but necessarily limits real-world generalisability [12]. As more malabsorptive procedures, particularly biliopancreatic diversion with duodenal switch (BPD/DS), and to a lesser degree OAGB, carry higher deficiency risk than SG or RYGB, the trajectories reported here likely represent a conservative estimate of the nutritional burden across the wider spectrum of MBS [25].

### Limitations

Several limitations apply. This was a single-centre retrospective cohort, introducing selection and referral bias. More importantly, informative attrition is likely. Numbers fell markedly at later timepoints, partly because recruitment continued until December 2025, so patients operated more recently had not yet reached the 5- and 10-year points, and partly through loss to follow-up. The later timepoints therefore reflect both an earlier-operated and a self-selected survivor subset, who may differ systematically from the whole cohort. Our models assume data are missing at random conditional on the covariates. If missingness instead reflects unobserved health status, later estimates may be biased, most plausibly underestimating deficiency. Reliance on routine clinical bloods introduces ascertainment and measurement bias: testing was incomplete, denominators varied by marker and timepoint, and assays or reference ranges may have shifted across 14 years. Procedure choice was not randomised, so residual confounding by indication persists despite adjustment. The breadth of testing raises multiplicity; although we applied false-discovery-rate correction within families, the subgroup analyses remain hypothesis-generating. Adherence was patient-reported and is subject to reporting bias, likely overestimating compliance. Supplementation was recorded at patient level only. Differences in supplement type, dose, duration or adherence over time, or between procedures, may therefore have influenced the observed trajectories and could not be accounted for. Similarly, PPI use was recorded as a binary patient-level variable, which may not reflect duration or cumulative exposure, so the PPI subgroup findings should be interpreted with caution. Weight outcomes were summarised at a single follow-up point, and fixed timepoints may not capture individual weight trajectories or recurrent weight gain, which may be better identified by assessment at specific early and later postoperative timepoints [26]. The absence of a non-surgical comparator group means the observed changes cannot be attributed to surgery itself, as opposed to the natural course of severe obesity, weight loss, or ageing over the follow-up period. Similarly, near-universal supplementation left no unsupplemented comparator, precluding estimation of the counterfactual deficiency burden.

## 5. Conclusions

Within a structured follow-up programme, supplement compliance was high and frank deficiency was uncommon for most markers. Over up to 10 years after MBS, however, progressive biochemical changes emerged and persisted despite near-universal supplementation and monitoring. These were most consistently falling calcium with rising PTH, a declining haemoglobin, and a downward drift in several trace elements and albumin; notably, repleting vitamin D did not abolish the secondary hyperparathyroid drive. The breadth and duration of this real-world panel argue for indefinite, systematic surveillance after MBS, with vigilance in patients taking PPIs and those with higher baseline BMI. Future work should define the optimal long-term monitoring schedule and establish whether these biochemical changes translate into clinical events such as fracture. It should also test prospectively, ideally in multicentre cohorts with complete ascertainment, whether targeted supplementation or PPI review modifies the response.

## Figures and Tables

**Table 1:** Baseline and operative characteristics of the analysis cohort (*n* = 426), overall and by sex and age group.

Variable	Category	Total	Male	Female	Age < 50	Age ≥ 50
Patients	*n*	426	75	351	227	199
Sex	Female	351 (82.4)	0 (0.0)	351 (100.0)	193 (85.0)	158 (79.4)
	Male	75 (17.6)	75 (100.0)	0 (0.0)	34 (15.0)	41 (20.6)
Age at surgery (yr)	Median [IQR]	49.0 [40.0–55.0]	51.0 [43.0–60.0]	48.0 [40.0–54.0]	41.0 [36.0–46.0]	56.0 [52.0–60.0]
	<50	227 (53.3)	34 (45.3)	193 (55.0)	227 (100.0)	0 (0.0)
	≥50	199 (46.7)	41 (54.7)	158 (45.0)	0 (0.0)	199 (100.0)
	Missing	0	0	0	0	0
Operation	Sleeve gastrectomy	235 (55.2)	34 (45.3)	201 (57.3)	130 (57.3)	105 (52.8)
	Roux-en-Y gastric bypass	175 (41.1)	37 (49.3)	138 (39.3)	85 (37.4)	90 (45.2)
	Adjustable gastric band	12 (2.8)	4 (5.3)	8 (2.3)	10 (4.4)	2 (1.0)
	Other *	4 (0.9)	0 (0.0)	4 (1.1)	2 (0.9)	2 (1.0)
	Missing	0	0	0	0	0
Baseline BMI (kg/m^2^)	Median [IQR]	44.2 [39.9–49.6]	44.6 [39.2–49.6]	44.2 [40.0–49.7]	45.6 [40.4–51.8]	43.5 [39.0–47.9]
Obesity class, baseline	Overweight (25–29.9) **	2 (0.5)	0 (0.0)	2 (0.6)	0 (0.0)	2 (1.1)
	Class I (30–34.9)	26 (6.3)	4 (5.6)	22 (6.5)	12 (5.4)	14 (7.4)
	Class II (35–39.9)	76 (18.5)	16 (22.5)	60 (17.6)	38 (17.1)	38 (20.1)
	Class III (>=40)	307 (74.7)	51 (71.8)	256 (75.3)	172 (77.5)	135 (71.4)
	Missing	15	4	11	5	10
Current BMI (kg/m^2^)	Median [IQR]	34.4 [29.6–40.1]	33.3 [30.5–40.2]	34.5 [29.5–40.1]	34.5 [29.5–40.2]	33.9 [29.8–40.0]
Obesity class, current	Normal/overweight (<25)	28 (6.8)	3 (4.2)	25 (7.4)	12 (5.4)	16 (8.5)
	Overweight (25–29.9)	82 (19.9)	14 (19.4)	68 (20.0)	49 (22.0)	33 (17.5)
	Class I (30–34.9)	115 (27.9)	24 (33.3)	91 (26.8)	59 (26.5)	56 (29.6)
	Class II (35–39.9)	81 (19.7)	13 (18.1)	68 (20.0)	44 (19.7)	37 (19.6)
	Class III (>=40)	106 (25.7)	18 (25.0)	88 (25.9)	59 (26.5)	47 (24.9)
	Missing	14	3	11	4	10
Baseline weight (kg)	Median [IQR]	121.3 [107.8–138.5]	136.3 [122.3–158.8]	119.0 [105.1–135.7]	124.2 [110.8–142.3]	118.5 [102.5–130.5]
Current weight (kg)	Median [IQR]	94.1 [80.0–110.1]	103.6 [94.4–119.2]	91.6 [77.5–107.0]	95.6 [83.7–114.1]	91.3 [77.0–106.7]
% total weight loss	Median [IQR]	21.6 [12.2–31.1]	20.2 [12.0–29.0]	21.7 [12.3–31.7]	22.5 [14.3–32.1]	20.3 [10.5–29.7]
% excess weight loss (%EWL)	Median [IQR]	49.2 [27.9–72.6]	46.7 [24.9–69.8]	50.5 [28.1–72.9]	51.7 [30.4–73.7]	47.4 [20.5–70.8]
	Missing	18	4	14	5	13
%EWL >50%	Yes	245 (60.0)	40 (56.3)	205 (60.8)	135 (60.8)	110 (59.1)
	No	163 (40.0)	31 (43.7)	132 (39.2)	87 (39.2)	76 (40.9)
	Missing	18	4	14	5	13
Follow-up (yr)	Median [IQR]	7.0 [2.2–11.0]	9.0 [5.0–12.0]	7.0 [2.0–10.0]	7.0 [2.0–11.0]	8.0 [4.0–11.0]
SIMD decile	1–2 (most deprived)	86 (21.2)	17 (24.3)	69 (20.5)	45 (20.8)	41 (21.6)
	3–4	132 (32.5)	20 (28.6)	112 (33.3)	73 (33.8)	59 (31.1)
	5–6	76 (18.7)	12 (17.1)	64 (19.0)	40 (18.5)	36 (18.9)
	7–8	65 (16.0)	13 (18.6)	52 (15.5)	32 (14.8)	33 (17.4)
	9–10 (least deprived)	47 (11.6)	8 (11.4)	39 (11.6)	26 (12.0)	21 (11.1)
	Missing	20	5	15	11	9
Type 2 diabetes (pre-op)	Yes	99 (23.5)	40 (53.3)	59 (17.0)	39 (17.2)	60 (30.8)
	No	323 (76.5)	35 (46.7)	288 (83.0)	188 (82.8)	135 (69.2)
	Missing/unknown	4	0	4	0	4
T2D resolved (of those with T2D)	Yes	41 (41.4)	13 (32.5)	28 (47.5)	20 (51.3)	21 (35.0)
	No	58 (58.6)	27 (67.5)	31 (52.5)	19 (48.7)	39 (65.0)
Supplement use	Forceval—prescribed	386 (91.0)	67 (90.5)	319 (91.1)	206 (90.7)	180 (91.4)
	Forceval—compliant (of prescribed)	357 (92.7)	65 (97.0)	292 (91.8)	187 (90.8)	170 (95.0)
	Vitamin D/calcium—prescribed	277 (65.3)	47 (63.5)	230 (65.7)	148 (65.2)	129 (65.5)
	Vitamin D/calcium—compliant (of prescribed)	268 (97.1)	46 (97.9)	222 (96.9)	143 (96.6)	125 (97.7)
	Ferrous fumarate—prescribed	169 (39.9)	24 (32.4)	145 (41.4)	95 (41.9)	74 (37.6)
	Ferrous fumarate—compliant (of prescribed)	157 (92.9)	23 (95.8)	134 (92.4)	86 (90.5)	71 (95.9)
	Vitamin B12—prescribed	376 (88.9)	67 (90.5)	309 (88.5)	202 (89.4)	174 (88.3)
	Vitamin B12—compliant (of prescribed)	361 (96.0)	65 (97.0)	296 (95.8)	193 (95.5)	168 (96.6)
	Proton pump inhibitor—prescribed	277 (65.3)	44 (59.5)	233 (66.6)	148 (65.2)	129 (65.5)
	Proton pump inhibitor—compliant (of prescribed)	270 (97.5)	42 (95.5)	228 (97.9)	143 (96.6)	127 (98.4)
	Vitamin K-prescribed	10 (2.4)	2 (2.7)	8 (2.3)	2 (0.9)	8 (4.1)
	Vitamin K-compliant (of prescribed)	9 (90.0)	2 (100.0)	7 (87.5)	2 (100.0)	7 (87.5)

Values are n (%) unless stated; continuous variables median [IQR]. * Other (*n* = 4): three gastric band removals and one gastric bypass revision, performed before 2011. ** Two patients had a baseline BMI < 30 kg/m^2^: one revisional (band-to-bypass, BMI reflecting post-band weight loss) and one with a referral BMI of 41 kg/m^2^ who lost weight during mandatory pre-operative optimisation. BMI, body mass index; SG, sleeve gastrectomy; RYGB, Roux-en-Y gastric bypass; AGB, adjustable gastric band; T2DM, type 2 diabetes mellitus; SIMD, Scottish Index of Multiple Deprivation; IQR, interquartile range; %TWL, percentage total weight loss; %EWL, percentage excess weight loss.

**Table 2:** Bone, iron and B-vitamin markers over time: number measured, median [IQR] and percentage outside the reference range at each postoperative timepoint (pooled cohort).

Marker/Timepoint	n	Median [IQR]	% Below Range	% Above Range
Bone & mineral metabolism				
Vitamin D				
pre-op	297	40.0 [24.0–58.5]	66.3	-
6 months	127	54.0 [37.0–70.0]	44.1	-
1 year	253	49.0 [35.0–65.0]	52.2	-
5 years	196	47.5 [32.0–68.0]	52.0	-
10 years	125	55.0 [41.5–71.0]	40.8	-
Adjusted calcium				
pre-op	387	2.4 [2.3–2.5]	2.6	1.8
6 months	347	2.4 [2.3–2.5]	2.9	1.2
1 year	342	2.4 [2.3–2.4]	3.2	1.2
5 years	247	2.4 [2.3–2.4]	4.0	2.0
10 years	182	2.3 [2.3–2.4]	4.4	1.6
Phosphate				
pre-op	352	1.1 [1.0–1.2]	5.7	4.0
6 months	307	1.1 [1.0–1.3]	1.6	6.8
1 year	296	1.2 [1.1–1.3]	1.4	4.4
5 years	189	1.1 [0.9–1.2]	6.3	4.8
10 years	119	1.1 [1.0–1.2]	6.7	3.4
Magnesium				
pre-op	371	0.8 [0.8–0.9]	4.0	4.9
6 months	222	0.9 [0.8–0.9]	1.4	9.9
1 year	237	0.9 [0.8–0.9]	1.7	10.5
5 years	144	0.9 [0.8–0.9]	4.2	13.2
10 years	52	0.9 [0.8–1.1]	3.8	40.4
Parathyroid hormone				
pre-op	292	5.7 [4.2–7.5]	0.7	29.8
6 months	285	5.5 [4.2–7.1]	1.4	26.0
1 year	289	5.3 [4.1–6.7]	0.7	22.8
5 years	197	5.8 [4.4–7.8]	3.0	32.5
10 years	125	5.8 [4.3–7.9]	3.2	32.8
Alkaline phosphatase				
pre-op	398	80.0 [64.8–97.2]	5.0	6.0
6 months	260	81.0 [63.0–100.0]	6.9	9.2
1 year	267	77.0 [60.0–96.0]	6.4	9.7
5 years	216	83.0 [65.2–107.8]	6.0	13.9
10 years	176	87.0 [67.2–107.8]	6.8	13.6
Iron status & anaemia				
Haemoglobin				
pre-op	415	138.0 [129.0–146.0]	26.3	0.2
6 months	365	135.0 [128.0–142.0]	29.3	0.3
1 year	354	134.0 [128.0–141.0]	31.1	0.3
5 years	263	133.0 [126.0–141.0]	35.4	0.0
10 years	218	133.5 [125.0–140.0]	38.5	0.0
Ferritin				
pre-op	375	76.0 [38.0–138.0]	10.4	5.6
6 months	333	77.0 [47.5–144.5]	6.6	7.8
1 year	325	77.0 [38.0–138.5]	8.9	6.2
5 years	238	52.0 [26.5–98.5]	16.0	6.7
10 years	161	72.0 [35.5–137.5]	10.6	9.3
Iron				
pre-op	380	13.0 [9.0–16.0]	56.8	1.3
6 months	335	13.0 [10.0–17.0]	51.3	2.7
1 year	331	14.0 [11.0–19.0]	43.2	3.9
5 years	233	15.0 [11.2–19.0]	43.8	3.0
10 years	144	14.9 [10.2–19.0]	43.1	4.2
Transferrin				
pre-op	378	2.7 [2.4–3.0]	2.9	0.0
6 months	334	2.4 [2.2–2.7]	11.4	0.0
1 year	329	2.5 [2.2–2.7]	10.6	0.0
5 years	230	2.6 [2.4–2.9]	5.2	0.0
10 years	142	2.6 [2.4–2.9]	6.3	0.7
Transferrin saturation				
pre-op	368	18.0 [13.0–23.0]	59.0	1.9
6 months	324	21.0 [15.0–27.0]	41.7	2.2
1 year	319	22.0 [17.0–28.0]	34.8	2.8
5 years	221	21.0 [16.0–27.0]	45.7	3.2
10 years	135	20.0 [15.0–27.0]	45.9	3.0
B-vitamins & haematinics				
Vitamin B12				
pre-op	374	367.5 [281.0–518.2]	3.5	-
6 months	329	626.0 [432.5–1063.0]	2.1	-
1 year	328	725.5 [466.5–1127.5]	3.7	-
5 years	235	816.0 [550.0–1154.0]	4.7	-
10 years	167	860.0 [547.0–1381.0]	2.4	-
Folate				
pre-op	358	6.8 [4.9–10.1]	4.7	-
6 months	325	12.4 [7.8–16.8]	2.2	-
1 year	311	13.6 [8.2–17.1]	1.6	-
5 years	221	13.6 [6.3–18.1]	5.4	-
10 years	157	15.6 [9.0–20.0]	5.7	-
Vitamin B1 (thiamine)				
pre-op	334	401.0 [334.5–468.2]	7.8	-
6 months	284	417.0 [355.0–502.8]	5.6	-
1 year	288	421.0 [361.2–500.0]	3.1	-
5 years	198	456.5 [394.5–531.0]	2.5	-
10 years	123	441.0 [382.0–515.0]	6.5	-
Vitamin B2				
pre-op	166	1.9 [1.6–2.2]	0.0	-
6 months	151	1.8 [1.6–2.1]	0.0	-
1 year	168	1.8 [1.6–2.1]	0.0	-
5 years	188	1.9 [1.7–2.1]	0.0	-
10 years	127	2.1 [1.9–2.3]	0.0	-
Vitamin B6				
pre-op	333	429.0 [350.5–544.5]	3.0	0.0
6 months	274	581.5 [440.0–733.2]	0.7	0.0
1 year	282	548.5 [410.8–711.2]	0.4	0.0
5 years	191	565.0 [411.0–791.0]	2.1	0.0
10 years	126	624.0 [493.5–818.0]	1.6	0.0

Values are median [IQR]; percentage below or above the reference range shown where applicable. IQR, interquartile range; SG, sleeve gastrectomy; RYGB, Roux-en-Y gastric bypass.

**Table 3:** Multivariable linear mixed-effects models of change per year for each marker, with sleeve-versus-bypass interaction, adjusted for procedure, sex, age, baseline BMI, preoperative diabetes and SIMD quintile.

Marker	Change per Year [95% CI]	*p* (FDR)	Sleeve × Time [95% CI]	*p* (FDR)	*n* obs/pts
Bone & mineral metabolism					
Vitamin D	1.195 [0.529, 1.862]	<0.001	−0.044 [−0.916, 0.829]	0.922	975/374
Adjusted calcium	−0.004 [−0.006, −0.002]	<0.001	0.002 [−0.001, 0.005]	0.324	1471/407
Phosphate	−0.007 [−0.012, −0.002]	0.009	−0.001 [−0.007, 0.006]	0.922	1232/383
Magnesium	0.008 [0.003, 0.014]	0.004	−0.004 [−0.011, 0.004]	0.645	1010/392
Parathyroid hormone	0.220 [0.105, 0.334]	<0.001	−0.189 [−0.339, −0.039]	0.081	1156/392
Alkaline phosphatase	1.173 [0.024, 2.322]	0.045	0.447 [−1.043, 1.937]	0.834	1294/402
Iron status & anaemia					
Haemoglobin	−0.322 [−0.569, −0.075]	0.026	−0.155 [−0.478, 0.168]	0.578	1575/408
Ferritin	4.168 [0.323, 8.013]	0.042	−3.774 [−8.746, 1.199]	0.342	1400/404
Iron	0.251 [0.039, 0.463]	0.034	−0.033 [−0.311, 0.245]	0.816	1390/402
Transferrin	0.009 [−0.002, 0.020]	0.113	0.004 [−0.010, 0.018]	0.714	1380/401
Transferrin saturation	0.460 [0.203, 0.717]	0.002	−0.309 [−0.645, 0.027]	0.342	1334/390
B-vitamins & haematinics					
Vitamin B12	29.082 [15.559, 42.604]	<0.001	16.349 [−1.257, 33.955]	0.172	1399/402
Folate	0.606 [0.432, 0.780]	<0.001	−0.320 [−0.544, −0.096]	0.026	1341/398
Vitamin B1	3.084 [−0.463, 6.631]	0.088	1.535 [−3.208, 6.279]	0.657	1197/388
Vitamin B2	0.033 [0.022, 0.044]	<0.001	−0.002 [−0.017, 0.014]	0.826	772/359
Vitamin B6	13.702 [6.378, 21.026]	<0.001	5.606 [−4.130, 15.342]	0.431	1173/394
Fat-soluble vitamins					
Vitamin A	0.019 [0.003, 0.035]	0.036	0.014 [−0.008, 0.035]	0.211	792/365
Vitamin E	−0.179 [−0.485, 0.126]	0.250	0.810 [0.388, 1.232]	<0.001	639/327
Trace elements					
Zinc	−0.075 [−0.129, −0.022]	0.015	0.074 [0.003, 0.146]	0.103	1134/382
Copper	−0.063 [−0.196, 0.070]	0.351	0.039 [−0.143, 0.221]	0.752	767/355
Selenium	−0.010 [−0.018, −0.002]	0.033	0.011 [−0.001, 0.022]	0.106	721/343
Red cell selenium	−0.097 [−0.155, −0.039]	0.005	0.087 [0.007, 0.168]	0.103	720/341
Manganese	−1.096 [−2.865, 0.672]	0.280	0.382 [−1.991, 2.755]	0.752	728/342
General/metabolic					
Albumin	−0.226 [−0.307, −0.146]	<0.001	−0.064 [−0.170, 0.043]	0.240	1345/387
Cholesterol	0.010 [−0.018, 0.038]	0.482	0.023 [−0.014, 0.059]	0.240	1133/375

Values are change per year [95% CI], adjusted for procedure, sex, age, baseline BMI, pre-operative T2DM and SIMD quintile. BMI, body mass index; T2DM, type 2 diabetes mellitus; SIMD, Scottish Index of Multiple Deprivation; PTH, parathyroid hormone; FDR, false discovery rate; CI, confidence interval.

## Data Availability

The data presented in this study are available on request from the corresponding author. The data are not publicly available due to privacy and information-governance restrictions.

## Supplementary Materials

The following supporting information can be downloaded at: https://www.mdpi.com/article/10.3390/nu18193265/s1, Table S1: Estimated change from the preoperative value at each timepoint for every marker; Table S2: Nutritional and metabolic markers over time by procedure; Table S3: Fat-soluble vitamins, trace elements, albumin and cholesterol over time; Table S4: Subgroup and sensitivity analyses; Figure S1: Fat-soluble vitamins by procedure; Figure S2: Trace elements by procedure; Figure S3: Albumin and total cholesterol by procedure.

## Abbreviations

The following abbreviations are used in this manuscript:

AGB	adjustable gastric band
BMI	body mass index
BOMSS	British Obesity and Metabolic Surgery Society
BPD/DS	biliopancreatic diversion with duodenal switch
%EWL	percentage excess weight loss
FDR	false discovery rate
IQR	interquartile range
MBS	metabolic and bariatric surgery
OAGB	one-anastomosis gastric bypass
PPI	proton pump inhibitor
PTH	parathyroid hormone
RYGB	Roux-en-Y gastric bypass
SG	sleeve gastrectomy
SHPT	secondary hyperparathyroidism
SIMD	Scottish Index of Multiple Deprivation
T2DM	type 2 diabetes mellitus
%TWL	percentage total weight loss
