# Supplementary material for: Nutritional and Metabolic Trajectories up to 10 Years After Metabolic and Bariatric Surgery: A Tertiary-Centre Retrospective Longitudinal Cohort Study
Source: Nutrients. 2026 Oct 3;18(19):3265. doi: 10.3390/nu18193265 (PMC13666773; doi:10.3390/nu18193265)

Supplementary Files

*[**Supplementary Table S1**. Estimated change from the preoperative value at each timepoint for every marker, from mixed models with time as a categorical factor (adjusted; FDR-corrected within marker family).]*

From mixed models with timepoint as a categorical factor, adjusted for procedure, sex, age, baseline BMI, diabetes and SIMD quintile. Estimated difference from pre-op [95% CI]; Benjamini-Hochberg FDR-adjusted p within family.

Marker / timepoint	Δ vs pre-op [95% CI]	p (FDR)
Bone & mineral metabolism		
Vitamin D		
6 months	7.37 [1.61, 13.12]	0.020
1 year	6.63 [2.05, 11.21]	0.005
5 years	8.19 [3.18, 13.20]	0.002
10 years	14.66 [8.73, 20.59]	<0.001
Adjusted calcium		
6 months	-0.00 [-0.02, 0.01]	0.959
1 year	-0.02 [-0.03, 0.00]	0.083
5 years	-0.01 [-0.03, 0.01]	0.428
10 years	-0.03 [-0.05, -0.01]	<0.001
Phosphate		
6 months	0.07 [0.04, 0.11]	<0.001
1 year	0.07 [0.03, 0.10]	<0.001
5 years	-0.03 [-0.07, 0.01]	0.336
10 years	-0.01 [-0.06, 0.03]	0.842

Marker / timepoint	Δ vs pre-op [95% CI]	p (FDR)
Magnesium		
6 months	0.02 [-0.01, 0.05]	0.285
1 year	0.04 [0.01, 0.07]	0.009
5 years	0.02 [-0.02, 0.06]	0.512
10 years	0.10 [0.04, 0.15]	<0.001
Parathyroid hormone		
6 months	-0.42 [-1.21, 0.37]	0.721
1 year	-0.43 [-1.22, 0.37]	0.577
5 years	-0.19 [-1.09, 0.71]	0.927
10 years	1.06 [-0.00, 2.12]	0.061
Alkaline phosphatase		
6 months	1.97 [-6.96, 10.90]	0.959
1 year	-2.04 [-10.91, 6.82]	0.909
5 years	10.40 [0.80, 19.99]	0.086
10 years	12.47 [2.13, 22.81]	0.017
Iron status & anaemia		
Haemoglobin		
6 months	-1.95 [-3.84, -0.07]	0.066
1 year	-2.63 [-4.54, -0.73]	0.003
5 years	-4.22 [-6.33, -2.11]	<0.001
10 years	-4.80 [-7.06, -2.53]	<0.001

Marker / timepoint	Δ vs pre-op [95% CI]	p (FDR)
Ferritin		
6 months	7.11 [-19.98, 34.20]	0.877
1 year	2.64 [-24.75, 30.04]	0.988
5 years	3.60 [-26.74, 33.95]	0.981
10 years	25.34 [-9.56, 60.24]	0.285
Iron		
6 months	0.93 [-0.47, 2.34]	0.372
1 year	2.99 [1.57, 4.41]	<0.001
5 years	2.70 [1.09, 4.31]	<0.001
10 years	2.82 [0.90, 4.74]	0.002
Transferrin		
6 months	-0.29 [-0.36, -0.22]	<0.001
1 year	-0.25 [-0.31, -0.18]	<0.001
5 years	-0.06 [-0.14, 0.02]	0.202
10 years	-0.06 [-0.15, 0.04]	0.387
Transferrin saturation		
6 months	2.97 [1.28, 4.65]	<0.001
1 year	4.20 [2.50, 5.89]	<0.001
5 years	3.76 [1.83, 5.70]	<0.001
10 years	4.17 [1.85, 6.49]	<0.001
B-vitamins & haematinics		

Marker / timepoint	Δ vs pre-op [95% CI]	p (FDR)
Vitamin B12		
6 months	369.91 [276.40, 463.41]	<0.001
1 year	406.88 [313.17, 500.59]	<0.001
5 years	440.88 [336.70, 545.06]	<0.001
10 years	559.16 [441.63, 676.70]	<0.001
Folate		
6 months	3.76 [2.62, 4.90]	<0.001
1 year	4.69 [3.52, 5.86]	<0.001
5 years	4.96 [3.65, 6.27]	<0.001
10 years	5.80 [4.31, 7.28]	<0.001
Vitamin B1		
6 months	15.83 [-9.42, 41.07]	0.435
1 year	18.64 [-6.51, 43.78]	0.266
5 years	51.43 [22.99, 79.87]	<0.001
10 years	33.27 [-0.20, 66.74]	0.052
Vitamin B2		
6 months	-0.01 [-0.10, 0.09]	0.995
1 year	-0.03 [-0.12, 0.07]	0.853
5 years	0.09 [-0.01, 0.19]	0.102
10 years	0.31 [0.20, 0.42]	<0.001
Vitamin B6		

Marker / timepoint	Δ vs pre-op [95% CI]	p (FDR)
6 months	139.38 [88.69, 190.07]	<0.001
1 year	144.04 [93.76, 194.32]	<0.001
5 years	164.79 [107.60, 221.98]	<0.001
10 years	228.99 [162.59, 295.39]	<0.001
Fat-soluble vitamins		
Vitamin A		
6 months	-0.25 [-0.37, -0.12]	<0.001
1 year	-0.07 [-0.20, 0.05]	0.440
5 years	0.05 [-0.09, 0.18]	0.782
10 years	0.16 [0.01, 0.31]	0.081
Vitamin E		
6 months	-0.67 [-2.99, 1.65]	0.844
1 year	-1.49 [-3.88, 0.91]	0.440
5 years	-4.57 [-7.13, -2.00]	<0.001
10 years	2.08 [-0.74, 4.89]	0.215
Trace elements		
Zinc		
6 months	-0.86 [-1.24, -0.47]	<0.001
1 year	-0.79 [-1.18, -0.41]	<0.001
5 years	-0.67 [-1.10, -0.24]	0.002
10 years	-0.76 [-1.26, -0.27]	0.002

Marker / timepoint	Δ vs pre-op [95% CI]	p (FDR)
Copper		
6 months	-1.70 [-2.78, -0.62]	<0.001
1 year	-2.19 [-3.27, -1.12]	<0.001
5 years	-1.40 [-2.53, -0.27]	0.015
10 years	-1.57 [-2.85, -0.29]	0.012
Selenium		
6 months	-0.15 [-0.21, -0.08]	<0.001
1 year	-0.13 [-0.19, -0.06]	<0.001
5 years	-0.12 [-0.19, -0.05]	<0.001
10 years	-0.12 [-0.20, -0.04]	0.002
Red cell selenium		
6 months	-0.36 [-0.86, 0.13]	0.267
1 year	-0.49 [-0.99, 0.00]	0.065
5 years	-0.38 [-0.88, 0.12]	0.245
10 years	-0.76 [-1.32, -0.19]	0.006
Manganese		
6 months	-0.88 [-15.42, 13.66]	0.996
1 year	-7.06 [-21.40, 7.28]	0.548
5 years	-2.07 [-17.60, 13.45]	0.975
10 years	-12.20 [-29.19, 4.78]	0.236
General / metabolic		

Marker / timepoint	Δ vs pre-op [95% CI]	p (FDR)
Albumin		
6 months	-1.84 [-2.40, -1.29]	<0.001
1 year	-1.82 [-2.39, -1.26]	<0.001
5 years	-2.57 [-3.21, -1.93]	<0.001
10 years	-3.37 [-4.09, -2.65]	<0.001
Cholesterol		
6 months	-0.13 [-0.31, 0.04]	0.187
1 year	-0.17 [-0.35, 0.00]	0.054
5 years	0.11 [-0.10, 0.33]	0.476
10 years	0.11 [-0.14, 0.36]	0.623

Bold = FDR p<0.05.

[**Supplementary Table S2**. *Nutritional and metabolic markers over time by procedure: number measured, median [IQR] and percentage outside the reference range at each timepoint.*]

Marker / timepoint	SG				RYGB			
	n	Median [IQR]	% below range	% above range	*n*	Median [IQR]	% below range	% above range
Bone and mineral metabolism								
Vitamin D (nmol/L)								
pre-op	168	40.5 [25.8-61.2]	66.7	-	120	40.0 [23.0-55.5]	65.8	-
6 months	70	55.0 [39.0-69.8]	41.4	-	53	46.0 [27.0-65.0]	50.9	-
1 year	133	50.0 [37.0-65.0]	48.9	-	113	44.0 [32.0-65.0]	57.5	-
5 years	108	47.0 [36.5-68.2]	53.7	-	86	49.5 [28.2-64.8]	50.0	-
10 years	77	56.0 [42.0-71.0]	35.1	-	47	49.0 [42.0-69.0]	51.1	-
Adjusted calcium (mmol/L)								
pre-op	219	2.40 [2.32-2.46]	1.8	2.3	158	2.38 [2.31-2.45]	3.8	1.3
6 months	191	2.40 [2.34-2.46]	2.1	0.0	146	2.36 [2.29-2.42]	4.1	2.7
1 year	184	2.38 [2.34-2.44]	1.6	0.5	149	2.37 [2.29-2.43]	5.4	1.3
5 years	134	2.40 [2.33-2.45]	2.2	3.0	110	2.35 [2.29-2.42]	5.5	0.9
10 years	105	2.37 [2.31-2.41]	3.8	2.9	75	2.30 [2.26-2.38]	5.3	0.0
Phosphate (mmol/L)								
pre-op	199	1.08 [0.95-1.19]	5.0	4.0	144	1.09 [0.98-1.18]	6.9	4.2
6 months	172	1.13 [1.04-1.27]	1.7	3.5	125	1.14 [1.04-1.30]	1.6	12.0
1 year	159	1.17 [1.05-1.25]	1.9	1.9	128	1.16 [1.06-1.29]	0.8	7.8

Marker / timepoint	SG				RYGB			
	n	Median [IQR]	% below range	% above range	*n*	Median [IQR]	% below range	% above range
5 years	104	1.08 [0.94-1.19]	6.7	4.8	83	1.10 [0.98-1.24]	6.0	4.8
10 years	69	1.09 [0.96-1.17]	5.8	2.9	49	1.09 [1.00-1.24]	8.2	4.1
Magnesium (mmol/L)								
pre-op	209	0.850 [0.810-0.890]	3.8	4.8	153	0.840 [0.800-0.880]	4.6	4.6
6 months	131	0.860 [0.820-0.910]	1.5	9.9	89	0.860 [0.820-0.910]	1.1	10.1
1 year	139	0.860 [0.815-0.920]	2.2	10.8	95	0.860 [0.810-0.905]	1.1	10.5
5 years	80	0.865 [0.808-0.903]	2.5	12.5	63	0.860 [0.790-0.925]	6.3	14.3
10 years	28	0.925 [0.850-1.078]	7.1	39.3	23	0.920 [0.830-1.090]	0.0	39.1
Parathyroid hormone (pmol/L)								
pre-op	171	5.80 [4.30-7.45]	0.6	29.2	111	5.70 [4.30-7.50]	0.9	31.5
6 months	165	5.40 [4.20-6.80]	2.4	23.6	110	5.65 [4.53-7.28]	0.0	30.0
1 year	155	5.10 [4.00-6.30]	0.0	18.7	126	5.65 [4.30-7.30]	1.6	29.4
5 years	108	5.55 [4.38-7.35]	0.9	29.6	87	6.00 [4.45-7.95]	5.7	35.6
10 years	72	5.35 [4.12-7.72]	1.4	33.3	51	6.20 [4.65-8.25]	5.9	33.3
Alkaline phosphatase (U/L)								
pre-op	224	79.5 [66.0-94.0]	2.7	4.0	163	80.0 [64.5-102.5]	8.0	9.2
6 months	151	80.0 [63.0-96.0]	5.3	6.0	106	82.5 [61.2-105.0]	9.4	14.2
1 year	151	74.0 [60.0-90.5]	3.3	6.0	113	82.0 [62.0-103.0]	10.6	14.2
5 years	118	80.0 [65.0-96.0]	3.4	11.0	95	89.0 [66.0-114.5]	9.5	15.8

Marker / timepoint	SG				RYGB			
	n	Median [IQR]	% below range	% above range	*n*	Median [IQR]	% below range	% above range
10 years	104	86.0 [66.8-107.0]	6.7	12.5	69	87.0 [68.0-107.0]	7.2	14.5
Iron status and anaemia								
Haemoglobin (g/L)								
pre-op	228	139.0 [128.0-147.2]	27.6	0.4	172	136.5 [130.0-144.0]	23.8	0.0
6 months	208	136.0 [129.0-144.0]	26.0	0.5	147	133.0 [127.5-139.0]	34.7	0.0
1 year	193	135.0 [129.0-142.0]	28.5	0.0	152	133.5 [127.0-140.0]	33.6	0.0
5 years	149	133.0 [126.0-140.0]	35.6	0.0	111	133.0 [127.0-141.0]	33.3	0.0
10 years	126	134.0 [127.0-140.8]	38.1	0.0	89	133.0 [124.0-139.0]	38.2	0.0
Ferritin (µg/L)								
pre-op	212	74.0 [36.5-137.2]	11.3	5.2	153	76.0 [40.0-133.0]	9.8	5.2
6 months	187	74.0 [47.0-141.5]	7.5	7.5	136	76.0 [47.0-137.0]	5.9	7.4
1 year	180	76.5 [38.8-141.5]	7.2	6.1	136	74.0 [33.0-124.0]	11.8	5.1
5 years	136	50.0 [25.0-87.5]	17.6	6.6	100	57.0 [27.0-122.0]	13.0	7.0
10 years	97	69.0 [32.0-131.0]	11.3	10.3	63	100.0 [44.0-151.0]	7.9	7.9
Iron (µmol/L)								
pre-op	216	12.5 [9.0-16.0]	56.9	1.4	155	12.0 [9.0-16.0]	58.1	1.3
6 months	188	13.0 [10.0-17.0]	50.5	2.7	137	13.0 [10.0-17.0]	53.3	2.2
1 year	182	14.0 [11.0-18.0]	45.6	3.3	140	15.0 [11.0-19.0]	41.4	4.3
5 years	129	14.0 [11.5-19.0]	44.2	2.3	101	15.0 [11.0-19.0]	42.6	3.0

Marker / timepoint	SG				RYGB			
	n	Median [IQR]	% below range	% above range	*n*	Median [IQR]	% below range	% above range
10 years	86	14.0 [11.0-19.0]	43.0	1.2	56	15.0 [10.8-21.0]	42.9	8.9
Transferrin (g/L)								
pre-op	216	2.70 [2.46-2.94]	3.7	0.0	153	2.69 [2.41-2.95]	2.0	0.0
6 months	190	2.41 [2.16-2.64]	10.5	0.0	134	2.43 [2.18-2.70]	12.7	0.0
1 year	182	2.45 [2.19-2.66]	8.2	0.0	138	2.50 [2.21-2.75]	14.5	0.0
5 years	127	2.67 [2.40-2.91]	3.9	0.0	100	2.59 [2.35-2.91]	7.0	0.0
10 years	85	2.62 [2.37-2.91]	5.9	0.0	55	2.59 [2.40-2.94]	7.3	1.8
Transferrin saturation (%)								
pre-op	214	18.0 [13.0-23.0]	58.9	1.4	145	17.0 [13.0-22.0]	60.0	2.8
6 months	186	21.0 [15.0-28.0]	41.4	2.2	128	21.0 [15.0-26.0]	44.5	1.6
1 year	177	22.0 [18.0-29.0]	36.7	2.8	133	22.0 [16.0-28.0]	33.8	2.3
5 years	122	21.5 [16.0-26.0]	45.9	2.5	96	21.5 [16.0-27.0]	43.8	4.2
10 years	80	20.0 [15.0-26.2]	45.0	0.0	53	21.0 [14.0-29.0]	45.3	7.5
B-vitamins and haematinics								
Vitamin B12 (ng/L)								
pre-op	211	358.0 [290.0-485.0]	2.4	-	153	380.0 [269.0-549.0]	5.2	-
6 months	181	655.0 [453.0-1018.0]	2.2	-	138	575.0 [402.8-1112.5]	2.2	-
1 year	180	769.0 [527.5-1153.0]	2.2	-	139	667.0 [390.5-1133.5]	5.8	-
5 years	130	864.5 [609.8-1157.0]	1.5	-	102	757.5 [455.5-1118.8]	8.8	-

Marker / timepoint	SG				RYGB			
	n	Median [IQR]	% below range	% above range	*n*	Median [IQR]	% below range	% above range
10 years	99	949.0 [659.5-1390.0]	1.0	-	66	699.0 [508.5-1264.0]	4.5	-
Folate (μg/L)								
pre-op	204	6.60 [4.80-9.90]	5.9	-	146	6.90 [5.12-10.88]	3.4	-
6 months	182	12.9 [7.9-16.4]	3.3	-	133	12.0 [7.7-16.0]	0.8	-
1 year	173	13.8 [8.3-17.1]	2.3	-	130	12.6 [8.1-16.9]	0.8	-
5 years	128	13.4 [5.7-17.2]	7.8	-	90	14.6 [8.4-20.0]	2.2	-
10 years	94	14.3 [6.3-20.0]	7.4	-	61	17.1 [10.7-20.0]	3.3	-
Thiamine, B1 (nmol TDP/g Hb)								
pre-op	188	400.0 [333.0-467.0]	8.5	-	136	411.5 [348.2-475.8]	6.6	-
6 months	160	410.5 [359.0-471.8]	7.5	-	114	438.0 [359.5-526.0]	2.6	-
1 year	152	421.5 [352.8-501.0]	4.6	-	129	421.0 [371.0-500.0]	0.8	-
5 years	114	448.0 [391.0-513.5]	2.6	-	82	473.5 [401.2-551.8]	2.4	-
10 years	68	426.5 [381.2-494.8]	5.9	-	54	465.5 [399.0-532.5]	7.4	-
Vitamin B2 (nmol FAD/g Hb)								
pre-op	82	1.80 [1.60-2.10]	0.0	-	75	1.90 [1.60-2.15]	0.0	-
6 months	78	1.90 [1.60-2.10]	0.0	-	65	1.80 [1.60-2.30]	0.0	-
1 year	77	1.90 [1.70-2.10]	0.0	-	84	1.80 [1.60-2.10]	0.0	-
5 years	110	1.90 [1.70-2.10]	0.0	-	76	2.00 [1.70-2.20]	0.0	-
10 years	70	2.05 [1.80-2.30]	0.0	-	55	2.20 [1.95-2.40]	0.0	-

Marker / timepoint	SG				RYGB			
	n	Median [IQR]	% below range	% above range	*n*	Median [IQR]	% below range	% above range
Vitamin B6 (pmol/g Hb)								
pre-op	187	414.0 [341.0-523.0]	3.7	0.0	135	436.0 [352.5-560.0]	2.2	0.0
6 months	154	549.0 [456.8-703.0]	1.3	0.0	110	591.0 [434.8-735.8]	0.0	0.0
1 year	149	578.0 [436.0-717.0]	0.0	0.0	125	530.0 [389.0-674.0]	0.8	0.0
5 years	110	535.5 [401.2-757.5]	0.9	0.0	79	627.0 [452.0-842.5]	3.8	0.0
10 years	71	600.0 [493.0-850.0]	0.0	0.0	53	662.0 [542.0-778.0]	3.8	0.0
Fat-soluble vitamins								
Vitamin A (µmol/L)								
pre-op	85	1.80 [1.50-2.20]	2.4	2.4	75	1.70 [1.40-2.00]	1.3	4.0
6 months	76	1.60 [1.30-1.90]	3.9	1.3	69	1.50 [1.20-1.70]	7.2	0.0
1 year	82	1.70 [1.40-2.00]	0.0	2.4	86	1.55 [1.30-1.90]	1.2	2.3
5 years	112	1.80 [1.50-2.10]	0.9	0.9	81	1.80 [1.40-2.10]	3.7	3.7
10 years	74	1.90 [1.60-2.27]	4.1	6.8	52	1.80 [1.48-2.20]	5.8	5.8
Vitamin E (µmol/L)								
pre-op	75	28.0 [26.0-32.5]	1.3	0.0	66	25.0 [21.2-29.8]	4.5	3.0
6 months	60	30.0 [26.0-33.0]	6.7	3.3	55	24.0 [19.0-26.0]	10.9	0.0
1 year	49	28.0 [6.6-33.0]	26.5	4.1	62	22.0 [19.0-28.8]	16.1	0.0
5 years	85	22.0 [5.8-30.0]	40.0	2.4	67	20.0 [5.7-26.0]	31.3	0.0
10 years	70	30.0 [24.0-35.8]	4.3	7.1	50	18.5 [14.2-23.0]	16.0	0.0

Marker / timepoint	SG				RYGB			
	n	Median [IQR]	% below range	% above range	*n*	Median [IQR]	% below range	% above range
Trace elements								
Zinc (µmol/L)								
pre-op	171	11.7 [10.6-12.6]	0.0	0.6	116	12.0 [10.9-13.0]	0.0	0.0
6 months	151	11.1 [10.1-11.9]	0.0	0.0	110	10.9 [10.0-12.1]	0.9	0.9
1 year	149	11.0 [9.9-12.1]	0.7	0.0	119	10.7 [9.7-11.8]	0.8	1.7
5 years	110	11.0 [10.2-11.7]	0.0	0.9	81	11.0 [9.9-12.0]	1.2	1.2
10 years	71	10.8 [9.9-11.9]	0.0	2.8	56	10.5 [9.4-11.9]	1.8	0.0
Copper (µmol/L)								
pre-op	85	19.6 [17.2-21.8]	1.2	21.2	72	18.8 [16.8-21.6]	1.4	19.4
6 months	75	17.8 [16.5-19.5]	0.0	8.0	69	17.2 [15.2-19.5]	1.4	4.3
1 year	79	17.0 [14.6-18.8]	1.3	5.1	82	16.6 [14.9-19.2]	2.4	7.3
5 years	103	17.6 [15.7-19.8]	1.0	6.8	80	17.6 [15.2-20.9]	0.0	18.8
10 years	68	17.4 [16.4-19.6]	0.0	5.9	54	17.2 [15.3-19.6]	3.7	9.3
Selenium (µmol/L)								
pre-op	77	1.09 [0.96-1.20]	2.6	1.3	67	1.10 [0.96-1.27]	0.0	3.0
6 months	73	0.990 [0.920-1.100]	6.8	0.0	65	0.970 [0.840-1.040]	7.7	0.0
1 year	67	1.04 [0.91-1.15]	4.5	1.5	74	0.930 [0.815-1.060]	14.9	0.0
5 years	106	1.03 [0.85-1.21]	8.5	5.7	73	0.920 [0.770-1.080]	19.2	0.0
10 years	70	0.995 [0.850-1.115]	4.3	4.3	49	0.890 [0.730-1.010]	26.5	4.1

Marker / timepoint	SG				RYGB			
	n	Median [IQR]	% below range	% above range	*n*	Median [IQR]	% below range	% above range
Red-cell selenium (nmol/g Hb)								
pre-op	82	6.30 [5.53-7.40]	6.1	4.9	70	6.70 [5.83-7.47]	2.9	4.3
6 months	67	6.10 [5.40-7.00]	7.5	3.0	60	6.00 [5.40-7.00]	5.0	1.7
1 year	56	6.60 [5.40-7.22]	10.7	5.4	75	5.60 [4.85-6.20]	10.7	1.3
5 years	104	6.00 [5.10-7.00]	6.7	1.9	77	5.70 [4.80-6.70]	9.1	5.2
10 years	74	5.82 [4.95-7.00]	10.8	2.7	55	5.20 [4.60-5.70]	12.7	0.0
Manganese (nmol/L)								
pre-op	87	139.0 [106.5-175.0]	0.0	1.1	69	178.0 [130.0-219.0]	0.0	5.8
6 months	79	143.0 [113.5-188.5]	3.8	3.8	63	160.0 [135.5-196.5]	4.8	4.8
1 year	76	153.5 [120.5-181.0]	2.6	2.6	85	149.0 [122.0-182.0]	3.5	1.2
5 years	82	157.0 [124.0-189.0]	2.4	1.2	65	155.0 [125.0-202.0]	3.1	1.5
10 years	71	149.0 [118.5-174.0]	4.2	0.0	51	140.0 [117.5-173.5]	2.0	2.0
General and metabolic								
Albumin (g/L)								
pre-op	205	39.0 [37.0-42.0]	12.7	0.5	152	39.0 [37.0-41.2]	14.5	2.0
6 months	178	38.0 [36.0-39.0]	23.6	0.0	136	38.0 [35.0-40.0]	26.5	0.0
1 year	167	37.0 [35.0-39.0]	26.9	0.0	137	38.0 [36.0-39.0]	24.8	0.0
5 years	117	37.0 [35.0-39.0]	29.9	0.0	96	37.0 [35.0-39.0]	35.4	0.0
10 years	91	36.0 [34.0-38.0]	47.3	0.0	66	37.0 [34.0-39.0]	37.9	0.0

Marker / timepoint	SG				RYGB			
	n	Median [IQR]	% below range	% above range	*n*	Median [IQR]	% below range	% above range
Total cholesterol (mmol/L)								
pre-op	202	4.75 [4.12-5.40]	-	-	143	4.30 [3.80-5.10]	-	-
6 months	158	4.80 [4.20-5.47]	-	-	103	4.00 [3.50-4.70]	-	-
1 year	157	4.80 [4.20-5.40]	-	-	113	4.00 [3.50-4.80]	-	-
5 years	89	5.00 [4.40-5.50]	-	-	67	4.10 [3.70-5.00]	-	-
10 years	60	5.25 [4.38-5.70]	-	-	41	4.10 [3.70-4.70]	-	-

[***Supplementary Table S3***. *Fat-soluble vitamins, trace elements, albumin and cholesterol over time: number measured, median [IQR] and percentage outside the reference range at each postoperative timepoint (pooled cohort).]*

Values median [IQR]. % below / above reference range where applicable. Glutathione peroxidase shown for completeness (insufficient n for inference).

Marker / timepoint	*n*	Median [IQR]	% below range	% above range
Fat-soluble vitamins				
Vitamin A				
pre-op	169	1.8 [1.5-2.1]	2.4	3.0
6 months	153	1.5 [1.3-1.8]	5.9	0.7
1 year	175	1.7 [1.3-1.9]	1.1	2.3
5 years	195	1.8 [1.4-2.1]	2.6	2.1
10 years	128	1.9 [1.5-2.2]	4.7	6.2
Vitamin E				
pre-op	149	27.0 [23.0-31.5]	2.7	2.0
6 months	123	26.0 [21.0-31.0]	8.9	1.6
1 year	118	25.0 [19.0-31.0]	20.3	1.7
5 years	154	21.0 [5.7-28.0]	36.4	1.3
10 years	122	24.0 [18.0-32.0]	9.8	4.1
Trace elements				
Zinc				
pre-op	298	11.8 [10.7-12.8]	0.0	0.7
6 months	271	11.1 [10.0-12.1]	0.4	0.4
1 year	276	10.9 [9.8-12.1]	0.7	0.7

Marker / timepoint	*n*	Median [IQR]	% below range	% above range
5 years	193	11.0 [10.0-11.9]	0.5	1.0
10 years	129	10.7 [9.6-11.9]	0.8	1.6
Copper				
pre-op	166	19.2 [16.8-21.6]	1.2	19.9
6 months	152	17.7 [15.5-19.5]	0.7	5.9
1 year	168	16.8 [14.6-18.9]	1.8	6.0
5 years	185	17.5 [15.4-19.9]	0.5	11.9
10 years	123	17.5 [15.5-19.6]	1.6	7.3
Selenium				
pre-op	153	1.1 [1.0-1.2]	1.3	2.6
6 months	146	1.0 [0.9-1.1]	6.8	0.0
1 year	148	1.0 [0.9-1.1]	10.1	0.7
5 years	181	1.0 [0.8-1.1]	12.7	3.3
10 years	121	0.9 [0.8-1.1]	14.0	4.1
Red cell selenium				
pre-op	161	6.6 [5.7-7.4]	4.3	4.3
6 months	135	6.1 [5.4-7.0]	5.9	2.2
1 year	137	5.9 [5.0-6.8]	10.2	2.9
5 years	182	5.9 [5.0-6.8]	7.7	3.8
10 years	131	5.5 [4.8-6.5]	11.5	1.5
Manganese				

Marker / timepoint	*n*	Median [IQR]	% below range	% above range
pre-op	165	150.0 [115.0-198.0]	0.0	3.6
6 months	150	151.0 [118.5-192.5]	4.0	4.0
1 year	168	149.5 [122.0-181.5]	3.0	1.8
5 years	148	155.0 [123.2-194.5]	2.7	1.4
10 years	124	144.5 [116.5-175.5]	4.0	0.8
General / nutritional status				
Albumin				
pre-op	367	39.0 [37.0-42.0]	13.4	1.1
6 months	324	38.0 [36.0-40.0]	24.4	0.0
1 year	313	38.0 [35.0-39.5]	25.2	0.0
5 years	216	37.0 [35.0-39.0]	31.9	0.0
10 years	159	36.0 [34.0-39.0]	42.8	0.0
Cholesterol				
pre-op	356	4.5 [4.0-5.3]	-	-
6 months	270	4.5 [3.8-5.2]	-	-
1 year	279	4.5 [3.7-5.2]	-	-
5 years	157	4.8 [4.0-5.3]	-	-
10 years	103	4.6 [3.8-5.4]	-	-

[**Supplementary Table S4**. Subgroup and sensitivity analyses: change per year for all 25 markers within each stratum (proton pump inhibitor use, adherence, gastrointestinal pathology, and baseline BMI <50 vs ≥50 kg/m^2), adjusted and FDR-corrected within marker family.]

Time slope (change per year) from the adjusted mixed model, re-fitted within each stratum. Each cell: estimate [95% CI]; bold = FDR p<0.05. PPI and baseline-BMI strata are stratified analyses; compliant-only and GI-exclusion are sensitivity analyses. Compliant-only: patients recorded as compliant with all prescribed supplements (n=357).

Marker	PPI: yes	PPI: no	Compliant only	Excluding GI pathology	Baseline BMI <50	Baseline BMI ≥50
Bone & mineral metabolism						
Vitamin D	1.252 [0.303, 2.201]	1.163 [0.264, 2.062]	1.162 [0.470, 1.855]	1.359 [0.605, 2.113]	1.110 [0.319, 1.900]	1.590 [0.375, 2.806]
Adjusted calcium	-0.004 [-0.007, -0.001]	-0.004 [-0.007, -0.001]	-0.005 [-0.007, -0.003]	-0.004 [-0.006, -0.001]	-0.004 [-0.006, -0.001]	-0.006 [-0.009, -0.002]
Phosphate	-0.008 [-0.015, -0.001]	-0.005 [-0.013, 0.002]	-0.005 [-0.011, 0.000]	-0.006 [-0.012, -0.001]	-0.002 [-0.007, 0.004]	-0.020 [-0.030, -0.010]
Magnesium	0.013 [0.006, 0.019]	0.005 [-0.004, 0.014]	0.009 [0.003, 0.016]	0.009 [0.003, 0.016]	0.013 [0.007, 0.019]	-0.004 [-0.015, 0.007]
Parathyroid hormone	0.311 [0.130, 0.491]	0.127 [0.005, 0.248]	0.061 [-0.032, 0.155]	0.249 [0.116, 0.383]	0.100 [0.001, 0.199]	0.602 [0.251, 0.954]
Alkaline phosphatase	1.084 [-0.781, 2.950]	1.409 [0.252, 2.566]	1.391 [0.083, 2.699]	1.211 [-0.124, 2.545]	0.906 [-0.501, 2.312]	1.908 [-0.014, 3.830]
Iron status & anaemia						
Haemoglobin	-0.316 [-0.683, 0.051]	-0.323 [-0.645, -0.002]	-0.349 [-0.619, -0.080]	-0.428 [-0.704, -0.152]	-0.247 [-0.535, 0.040]	-0.538 [-1.024, -0.052]
Ferritin	3.620 [-1.861, 9.100]	4.589 [-0.684, 9.863]	4.369 [0.063, 8.675]	5.684 [1.185, 10.184]	6.409 [1.703, 11.116]	-1.164 [-7.399, 5.071]
Iron	0.291 [0.040, 0.542]	0.224 [-0.147, 0.595]	0.232 [-0.007, 0.472]	0.310 [0.062, 0.557]	0.252 [-0.022, 0.525]	0.287 [0.011, 0.563]
Transferrin	0.010 [-0.005, 0.026]	0.007 [-0.008, 0.022]	0.008 [-0.004, 0.020]	-0.001 [-0.012, 0.011]	-0.004 [-0.016, 0.007]	0.040 [0.016, 0.065]
Transferrin saturation	0.755 [0.406, 1.104]	0.099 [-0.274, 0.471]	0.573 [0.291, 0.856]	0.552 [0.258, 0.846]	0.242 [-0.049, 0.533]	1.027 [0.493, 1.560]
B-vitamins & haematinics						
Vitamin B12	29.319 [9.618, 49.019]	29.051 [10.723, 47.379]	27.656 [13.238, 42.074]	34.546 [19.449, 49.643]	30.237 [14.295, 46.180]	26.327 [0.787, 51.867]
Folate	0.548 [0.297, 0.799]	0.657 [0.424, 0.890]	0.714 [0.522, 0.905]	0.611 [0.412, 0.810]	0.649 [0.442, 0.855]	0.450 [0.146, 0.753]
Vitamin B1	2.144 [-3.012, 7.299]	4.507 [-0.140, 9.154]	4.283 [0.383, 8.184]	1.234 [-2.664, 5.132]	2.096 [-2.215, 6.407]	5.710 [-0.328, 11.749]

Marker	PPI: yes	PPI: no	Compliant only	Excluding GI pathology	Baseline BMI <50	Baseline BMI ≥50
Vitamin B2	0.025 [0.010, 0.040]	0.043 [0.025, 0.060]	0.034 [0.022, 0.046]	0.028 [0.015, 0.040]	0.033 [0.020, 0.046]	0.030 [0.009, 0.052]
Vitamin B6	13.865 [3.820, 23.910]	13.726 [2.930, 24.521]	15.594 [7.796, 23.392]	11.820 [3.555, 20.086]	15.992 [7.561, 24.423]	8.015 [-7.073, 23.102]
Fat-soluble vitamins						
Vitamin A	0.006 [-0.014, 0.026]	0.032 [0.006, 0.058]	0.020 [0.005, 0.036]	0.022 [0.005, 0.039]	0.025 [0.008, 0.042]	0.004 [-0.036, 0.045]
Vitamin E	-0.226 [-0.613, 0.160]	-0.049 [-0.584, 0.486]	-0.147 [-0.482, 0.188]	-0.240 [-0.570, 0.089]	-0.183 [-0.523, 0.158]	-0.011 [-0.733, 0.710]
Trace elements						
Zinc	-0.104 [-0.176, -0.033]	-0.052 [-0.134, 0.030]	-0.084 [-0.141, -0.027]	-0.077 [-0.138, -0.017]	-0.098 [-0.161, -0.034]	-0.014 [-0.112, 0.084]
Copper	-0.154 [-0.323, 0.015]	0.103 [-0.115, 0.321]	-0.087 [-0.233, 0.059]	-0.020 [-0.160, 0.120]	-0.069 [-0.224, 0.085]	-0.089 [-0.347, 0.168]
Selenium	-0.025 [-0.037, -0.013]	0.002 [-0.009, 0.013]	-0.011 [-0.021, -0.002]	-0.008 [-0.018, 0.001]	-0.006 [-0.015, 0.004]	-0.023 [-0.042, -0.003]
Red cell selenium	-0.098 [-0.170, -0.026]	-0.081 [-0.183, 0.021]	-0.114 [-0.175, -0.053]	-0.114 [-0.179, -0.049]	-0.102 [-0.166, -0.038]	-0.057 [-0.209, 0.095]
Manganese	-1.288 [-3.699, 1.123]	-0.892 [-3.565, 1.780]	-1.735 [-3.539, 0.069]	-1.285 [-3.196, 0.626]	-1.708 [-3.571, 0.156]	1.595 [-3.461, 6.650]
General / metabolic						
Albumin	-0.277 [-0.390, -0.163]	-0.167 [-0.280, -0.054]	-0.223 [-0.311, -0.135]	-0.226 [-0.313, -0.138]	-0.190 [-0.282, -0.097]	-0.316 [-0.481, -0.152]
Cholesterol	-0.024 [-0.062, 0.014]	0.041 [-0.001, 0.083]	0.012 [-0.019, 0.042]	-0.006 [-0.038, 0.026]	0.014 [-0.020, 0.048]	0.000 [-0.050, 0.051]

Bold = FDR p<0.05. Cells show change per year [95% CI]

[**Supplementary Figure S1**. Fat-soluble vitamins (vitamin A, vitamin E) by procedure across timepoints]

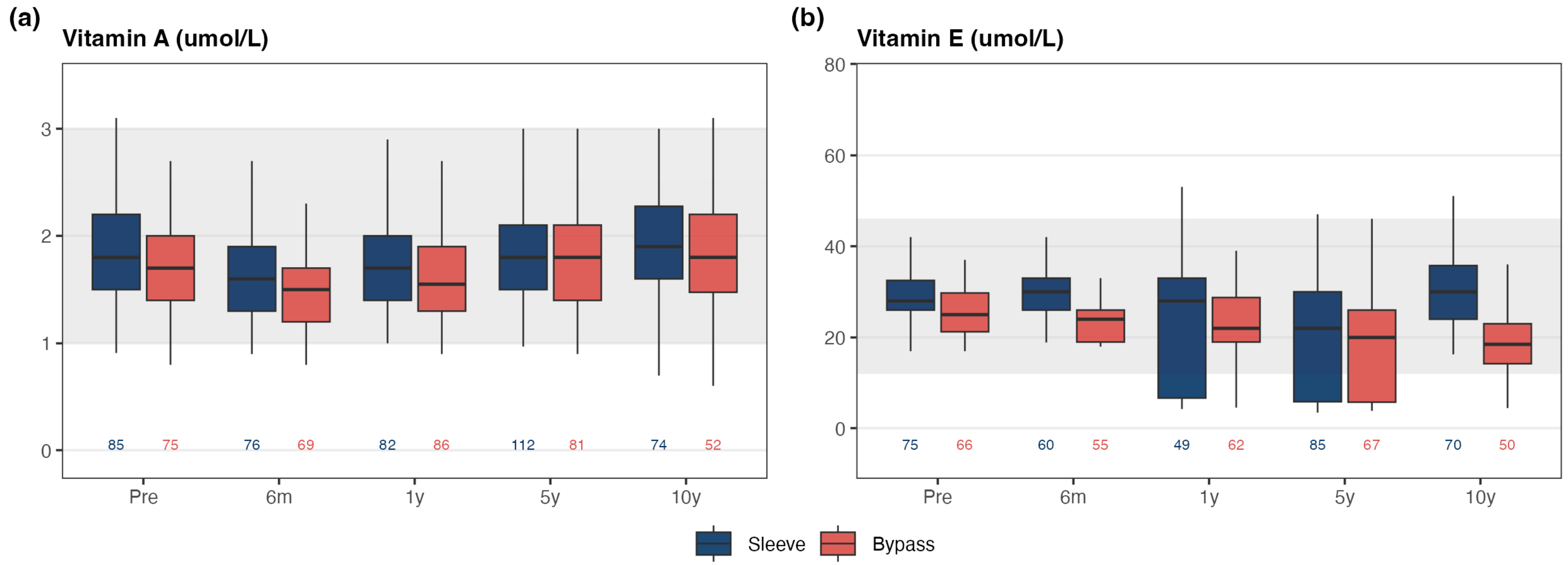

[**Supplementary Figure S2**. Trace elements (zinc, copper, selenium, red-cell selenium, manganese) by procedure across timepoints.]

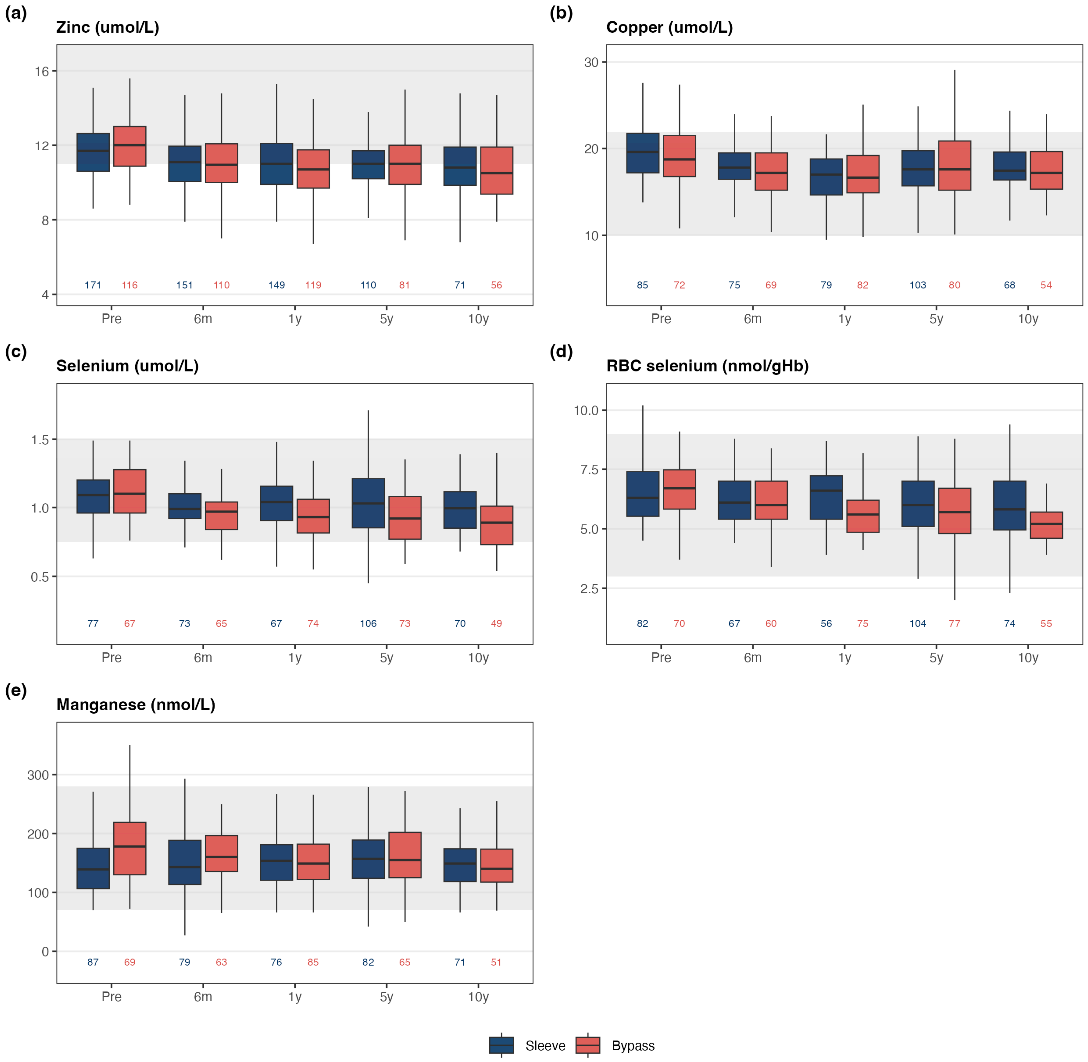

[**Supplementary Figure S3**. Albumin and total cholesterol by procedure across timepoints.]

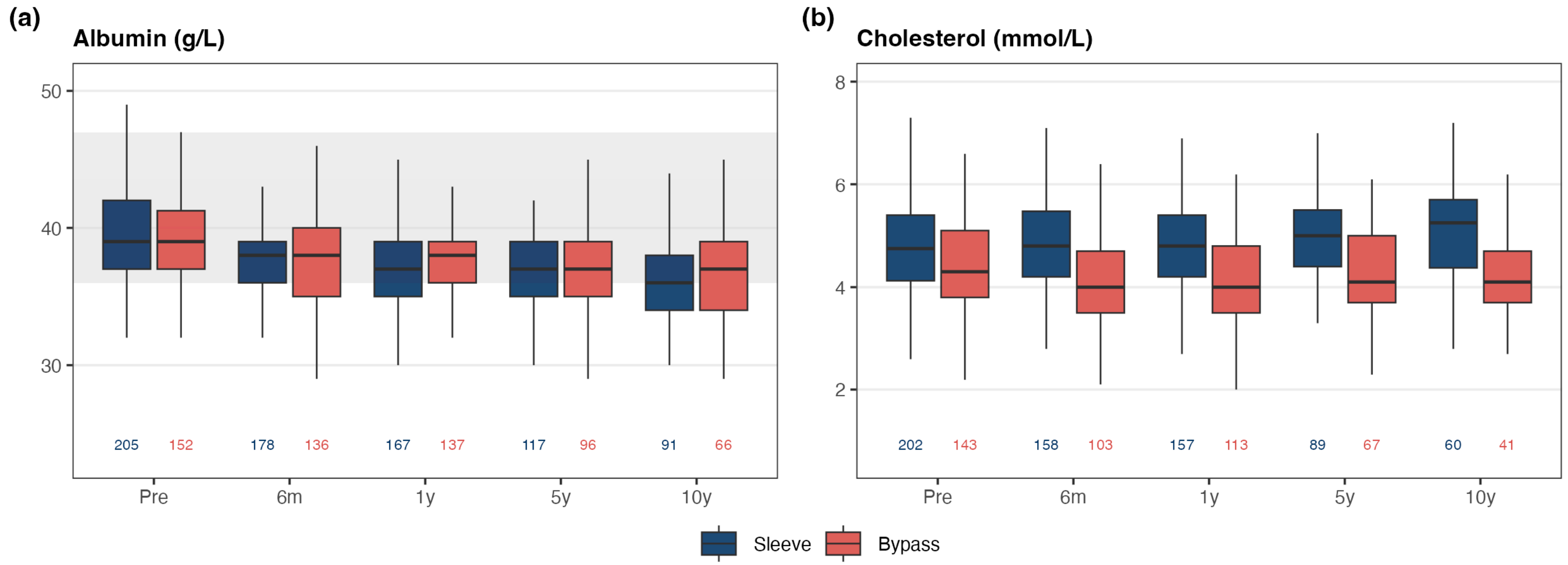

Supplement: Supplementary file 1 [file nutrients-18-03265-s001.zip › nutrients-4585148-supplementary.pdf]
